# Predictors of 6-month clinical change in primary progressive aphasia following language therapy and transcranial magnetic stimulation

**DOI:** 10.1093/braincomms/fcag398

**Published:** 2026-10-19

**Authors:** Lucía Fernández-Romero, Cristina Delgado-Alonso, Stephanie M Grasso, María Nieves Cabrera-Martín, Paloma Balugo, Adela Fraile-Pereda, Paz Suárez-Coalla, Yadhira Barroso, Silvia Oliver-Mas, Jorge Matias-Guiu, Javier Olazarán, María Díez-Cirarda, Jordi A Matias-Guiu

**Affiliations:** Department of Neurology, Hospital Clínico San Carlos, San Carlos Health Research Institute (IdISSC), Universidad Complutense de Madrid, Madrid 28040, Spain; Department of Neurology, Hospital Clínico San Carlos, San Carlos Health Research Institute (IdISSC), Universidad Complutense de Madrid, Madrid 28040, Spain; Department of Speech, Language and Hearing Sciences, University of Texas, Austin, TX 78712, USA; Department of Nuclear Medicine, Hospital Clínico San Carlos, San Carlos Health Research Institute (IdISSC), Universidad Complutense de Madrid, Madrid 28040, Spain; Department of Clinical Neurophysiology, Hospital Clínico San Carlos, San Carlos Health Research Institute (IdISSC), Universidad Complutense de Madrid, Madrid 28040, Spain; Department of Clinical Neurophysiology, Hospital Clínico San Carlos, San Carlos Health Research Institute (IdISSC), Universidad Complutense de Madrid, Madrid 28040, Spain; Faculty of Psychology, University of Oviedo, Oviedo 33009, Spain; Department of Neurology, Hospital Clínico San Carlos, San Carlos Health Research Institute (IdISSC), Universidad Complutense de Madrid, Madrid 28040, Spain; Department of Neurology, Hospital Clínico San Carlos, San Carlos Health Research Institute (IdISSC), Universidad Complutense de Madrid, Madrid 28040, Spain; Department of Neurology, Hospital Clínico San Carlos, San Carlos Health Research Institute (IdISSC), Universidad Complutense de Madrid, Madrid 28040, Spain; Department of Neurology, Hospital Gregorio Marañón, Gregorio Marañón Health Research Institute (IiSGM), Madrid 28007, Spain; Department of Neurology, Hospital Clínico San Carlos, San Carlos Health Research Institute (IdISSC), Universidad Complutense de Madrid, Madrid 28040, Spain; Department of Neurology, Hospital Clínico San Carlos, San Carlos Health Research Institute (IdISSC), Universidad Complutense de Madrid, Madrid 28040, Spain

**Keywords:** frontotemporal dementia, brain stimulation, neuromodulation, Alzheimer’s disease, neuroimaging

## Abstract

Primary progressive aphasia (PPA) is a neurodegenerative disorder characterized by progressive language impairment. Available treatments and information on factors associated with clinical change and treatment response remain limited. This study is a *post hoc* analysis of a randomized controlled trial and aimed to identify baseline demographic, cognitive and neuroimaging factors associated with clinical change in a subanalysis of a double-blind, randomized, parallel clinical trial of transcranial magnetic stimulation (TMS) and language therapy in PPA. Fifty-seven patients diagnosed with PPA received 6 months of lexical retrieval treatment, 40 with active TMS and 17 with sham TMS. All participants underwent comprehensive cognitive and language assessments before treatment and were evaluated using the Mini Linguistic State Examination (MLSE), naming task, words per minute, the Interview for Deterioration in Daily Living Activities in Dementia (IDDD), the Neuropsychiatric Inventory (NPI) and fluorodeoxyglucose-PET scans before and after treatment. Predictors of clinical change were analysed using least absolute shrinkage and selection operator (LASSO) and Elastic Net regression models, and unsupervised k-means clustering was applied to identify response profiles. Models for MLSE (LASSO: *R*2 = 0.52; Elastic Net: *R*^2^ = 0.57), naming (LASSO: *R*^2^ = 0.42; Elastic Net: *R*^2^ = 0.41), IDDD (LASSO: *R*^2^ = 0.46; Elastic Net: *R*^2^ = 0.58) and NPI (LASSO: *R*^2^ = 0.81; Elastic Net: *R*^2^ = 0.77) demonstrated good predictive accuracy. TMS treatment consistently emerged as the strongest predictor of clinical improvement across all outcomes. Higher metabolism in several brain regions (left hippocampus, posterior cingulate and orbitofrontal cortex) predicted greater clinical improvement. Cluster analysis identified two subgroups: responders and non-responders, with 77.5% of patients receiving active TMS in the responder cluster and 82.4% of the sham group in the non-responder cluster. Among patients receiving active TMS, a positive effect was associated with the non-fluent and semantic variants and with better cognitive performance in non-language domains. In conclusion, TMS treatment emerged as the main factor associated with clinical improvement on cognitive-linguistic and functional measures. Better cognitive performance and preserved brain metabolism in key regions were linked to more favourable clinical outcomes. These findings underscore the potential of TMS in combination with language therapy and pave the way for precision medicine approaches in PPA, emphasizing the need to identify individual profiles that predict optimal treatment response.

## Teaser

Fernandez-Romero *et al*. report a *post hoc* analysis of a clinical trial in primary progressive aphasia examining baseline predictors of clinical change following transcranial magnetic stimulation (TMS) and language therapy. TMS, cognitive performance and brain metabolism were associated with outcomes, with a more favourable response of non-fluent and semantic variants to TMS.

## Graphical Abstract

**Graphical Abstract:**
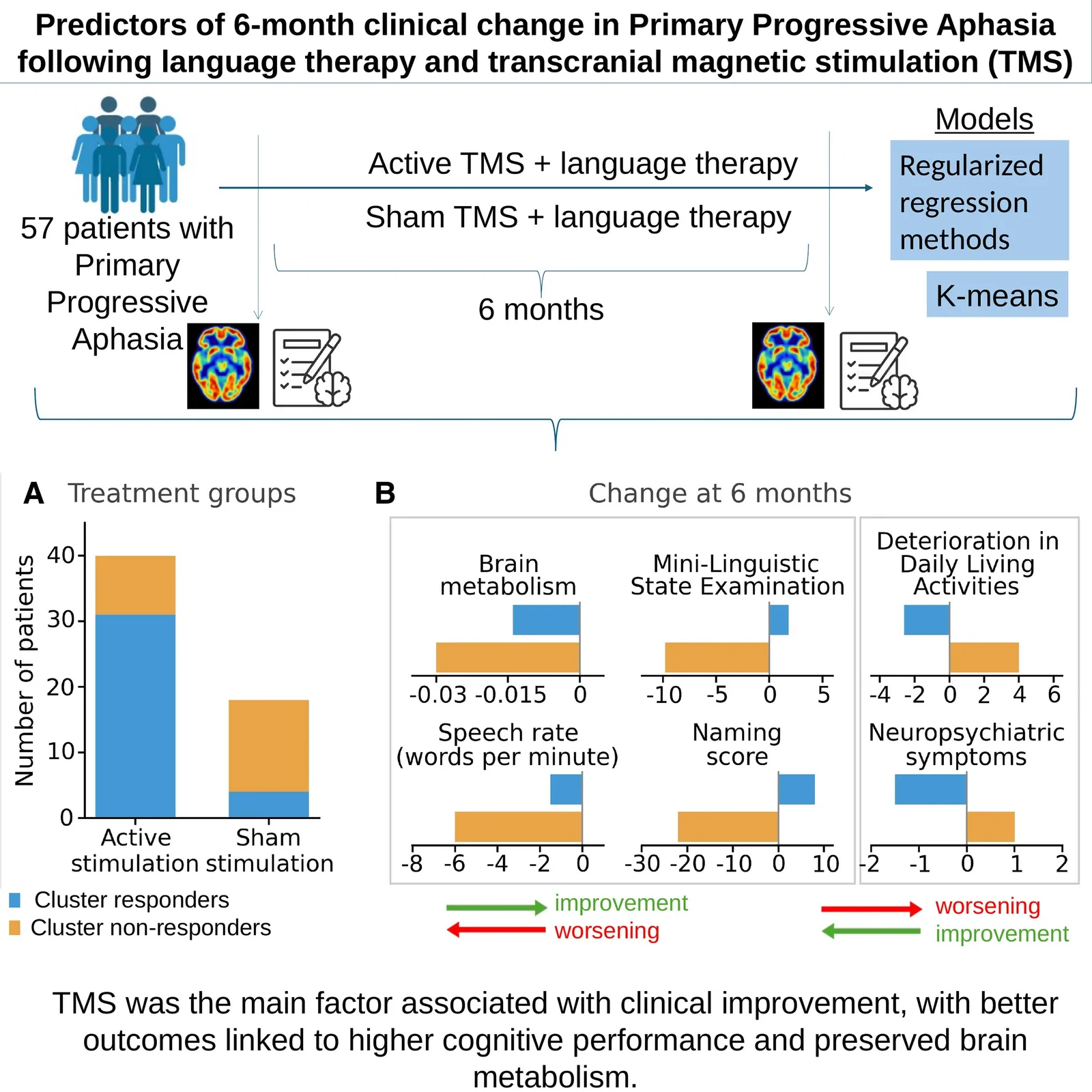

## Introduction

Primary progressive aphasia (PPA) is a neurodegenerative syndrome characterized by progressive language deterioration linked to several forms of frontotemporal degeneration as well as Alzheimer’s disease.^1,2^ Over the last few decades, there have been important advances in the clinical, neuroimaging and biological characterization of PPA.^3-5^ In this regard, three main clinical variants are recognized: non-fluent/agrammatic (nfvPPA), characterized by effortful, agrammatic speech and/or apraxia of speech; semantic (svPPA), characterized by progressive loss of semantic knowledge and word comprehension; and logopenic (lvPPA), characterized by impaired word retrieval and sentence repetition.^5^ These variants are associated with left frontal, anterior temporal and left parieto-temporal damage, respectively. Pathologically, the nfvPPA is mainly associated with tau followed by TDP-43 deposition, svPPA is closely linked to TDP-43 type C proteinopathy, and lvPPA is considered an atypical variant of Alzheimer’s disease.^2^ The approval of anti-amyloid monoclonal antibodies signals the onset of a new phase in the management of neurodegenerative diseases,^6^ with expectations of increasing access to effective pharmacological treatments in the near future.^7^

In the field of PPA, due to focal damage observed in the left hemisphere, one of the most innovative therapies is the application of non-invasive brain stimulation techniques.^8-10^ However, most studies have used crossover designs, applied brain stimulation for only a few weeks and included relatively small patient samples. We recently showed that a 6-month treatment of excitatory transcranial magnetic stimulation (TMS) over the left prefrontal dorsolateral cortex combined with language therapy mitigated the progression of the disease in a randomized, controlled, parallel and double-blind clinical trial conducted in 63 patients with the three variants of PPA.^11^ Specifically, in comparison to sham TMS, patients receiving active TMS showed improvement or less decline in language function, naming abilities, functional activities and neuropsychiatric symptoms. TMS was well tolerated, with minimal side effects and high adherence to the protocol combining brain stimulation and language therapy. Attrition rates were low and unrelated to the intervention, supporting the feasibility of this approach in patients with PPA.

One of the major challenges in current PPA research is the scarcity of longitudinal studies, which hinders both the understanding of disease progression and the development of targeted interventions. Most of the studies in PPA have been cross-sectional, and therefore limited information is available about factors associated with trajectories of progression. This information is relevant for guiding the design of future clinical trials in the field.

In this study, we analysed predictive factors associated with clinical progression over a 6-month period using data from our brain stimulation clinical trial. Specifically, we examined demographic, clinical, neuropsychological and fluorodeoxyglucose (FDG)-PET imaging variables to identify predictors of change in language abilities, neuropsychiatric symptoms and functional outcomes. We also explored whether predictors of clinical change differed across PPA variants. Additionally, we aimed to identify factors associated with response to TMS therapy using clustering analysis.

## Materials and methods

### Participants and study design

This is a *post hoc* analysis of a prospective, randomized, controlled, double-blind, parallel-group clinical trial investigating the effects of TMS combined with language therapy in individuals with PPA. The study was approved by the Ethics Committee of the Hospital Clinico San Carlos, following the Declaration of Helsinki and its later amendments. All patients signed written informed consent. Sixty-three participants meeting consensus criteria for PPA^11^ were randomly assigned to receive either active or sham TMS over the left dorsolateral prefrontal cortex (DLPFC) for 6 months, alongside language therapy. The DLPFC was selected, as described in the main clinical trial, based on previous evidence supporting its role in language-related networks and prior studies demonstrating therapeutic benefits in PPA.^11^ Further information on the inclusion and exclusion criteria, as well as the distribution of participants, is provided in Supplementary Tables 1 and 2. The TMS intervention followed an excitatory theta-burst protocol, starting with an intensive phase (10 sessions across 2 weeks) and continuing with a 22-week maintenance phase (1 session per week). Of the 63 participants initially enrolled, one participant (with lvPPA) in the sham group withdrew at 3 months due to caregiver-related difficulties in attending hospital visits, as the patient had mobility limitations and was reluctant to leave home. Two additional participants (one lvPPA and one nfvPPA) in the sham group were diagnosed with cancer during the study period and initiated chemotherapy, requiring withdrawal from the study. Three participants did not complete all intervention sessions (one with lvPPA, one with svPPA and one with nfvPPA), missing a small number of sessions due to medical reasons (ear surgery) or caregiver unavailability. In addition, three participants (two with svPPA and one with nfvPPA) declined the final PET scan and were therefore excluded due to missing post-treatment PET imaging data. We included in this analysis only those participants who completed all clinical and neuroimaging assessments throughout the entire follow-up period (*n* = 57) (Table 1). All participants were in relatively early stages of PPA and were able to engage in the intervention. The distribution of baseline scores across outcome measures is shown in Supplementary Fig. 1.

**Table 1:** Baseline clinical and demographic characteristics of the cohort, overall and by PPA variant

	Whole cohort (*n* = 57)	nfvPPA (*n* = 22)	svPPA (*n* = 10)	lvPPA (*n* = 25)
Sex (female)	38 (66.7%)	17 (77.3%)	6 (60%)	15 (60%)
Age	71.33 (8.49)	71.04 (9.13)	65.3 (9.15)	74 (6.42)
Years of education	13.53 (4.77)	13.68 (4.57)	13.50 (6.06)	13.40 (4.58)
Treatment (active TMS)	40 (70.2%)	16 (72.7%)	7 (70%)	17 (68%)
Baseline assessment				
SURV	0.801 (0.87)	0.81 (0.12)	0.84 (0.08)	0.78 (0.05)
ACE-III	52.90 (19.54)	61.23 (24.18)	56.40 (10.69)	44.16 (13.71)
MLSE total	78.60 (11.69)	82.77 (12.66)	80.40 (11.93)	74.20 (9.37)
Naming (/261)	146.12 (73.55)	191.64 (77.22)	109.20 (53.57)	120.84 (56.53)
WPM	66.71 (32.95)	48.90 (30.31)	97.09 (20.91)	66.67 (30.12)
IDDD	46.30 (12.98)	44.14 (14.05)	48.30 (13.32)	47.40 (12.12)
NPI	8.74 (12.42)	6.50 (9.78)	10.70 (11.07)	9.92 (14.91)
Digit span (forward)	4.46 (1.42)	4.32 (1.39)	5.50 (1.35)	4.16 (1.31)
Digit span (backward)	2.53 (1.31)	2.45 (1.63)	3.20 (0.92)	2.32 (1.07)
Corsi blocks (forward)	4.32 (1.48)	4.32 (1.56)	5.60 (0.84)	3.80 (1.32)
Corsi blocks (backward)	2.95 (1.70)	2.86 (1.91)	4.50 (1.51)	2.40 (1.16)
TMT-A (time)	148.61 (159.56)	191.09 (240.36)	77.20 (43.90)	139.80 (65.36)
TMT-B (time)	570.60 (350.12)	520.55 (368.22)	318.80 (315.13)	715.36 (282.14)
SDMT	16.47 (12.60)	18.23 (13.76)	28.30 (10.66)	10.20 (7.74)
ROCF copy	22.80 (11.01)	24.75 (10.94)	31.00 (4.37)	17.80 (10.64)
ROCF (time)	270.11 (179.93)	227.14 (176.86)	169.10 (85.38)	348.32 (181.98)
ROCF (3-min recall)	8.68 (6.66)	11.23 (7.13)	12.15 (6.03)	5.04 (4.53)
ROCF (30-min recall)	7.75 (6.85)	10.05 (6.94)	12.00 (7.18)	4.04 (4.63)
ROCF (recognition)	16.53 (5.45)	15.14 (8.03)	17.80 (2.86)	17.24 (2.60)
Stroop word	45.88 (20.64)	39.86 (17.02)	64.70 (24.96)	43.64 (17.91)
Stroop colour	23.67 (15.15)	25.00 (15.70)	35.70 (13.55)	17.68 (12.32)
Stroop interference	10.75 (11.81)	10.36 (11.50)	23.80 (13.01)	5.88 (7.11)
ToL total correct	1.37 (2.02)	1.50 (2.04)	3.50 (2.32)	0.40 (0.96)
VOSP object decision	14.84 (4.09)	14.55 (5.54)	15.80 (3.26)	14.72 (2.78)
VOSP progressive silhouettes	11.89 (4.83)	8.64 (4.71)	15.80 (3.91)	13.20 (3.38)
VOSP position discrimination	18.30 (3.08)	17.95 (4.32)	19.80 (0.42)	18.00 (2.16)
VOSP number location	7.09 (3.36)	7.27 (3.58)	8.50 (1.72)	6.36 (3.56)
Semantic fluency (animals)	6.21 (4.31)	7.82 (5.84)	5.00 (2.36)	5.28 (2.73)
Formal fluency (P)	6.11 (3.69)	5.36 (4.40)	7.70 (3.47)	6.12 (2.96)

Values are presented as mean (SD), unless otherwise indicated. Abbreviations: PPA, primary progressive aphasia; nfvPPA, non-fluent variant; svPPA, semantic variant; lvPPA, logopenic variant; TMS, transcranial magnetic stimulation; MLSE, Mini Linguistic State Examination; WPM, words per minute; IDDD, Interview for Deterioration in Daily Living Activities in Dementia; NPI, Neuropsychiatric Inventory; TMT, Trail Making Test; SDMT, Symbol Digit Modalities Test; ROCF, Rey–Osterrieth Complex Figure; ToL, Tower of London; VOSP, Visual Object and Space Perception Battery; ACE, Addenbrooke’s Cognitive Examination; CSC, complex sentence comprehension; VTA, verb tense agreement.

All participants were evaluated at baseline, after 3 months and at the end of the 6-month intervention using the Mini Linguistic State Examination (MLSE), words per minute (WPM) from spontaneous speech samples, a confrontation naming list, the Interview for Deterioration in Daily Living Activities in Dementia (IDDD) and the Neuropsychiatric Inventory (NPI). All participants underwent FDG-PET scan before and at the end of the treatment. Further details about the study design and procedures have been outlined in detail elsewhere, and we summarize the methodological details most relevant to the current study in the sections that follow.^10^

### Language therapy

Immediately after the 3-min TMS session, all participants received ∼50 min of language therapy. The intervention consisted of an adapted version of Lexical Retrieval Treatment (LRT).^12-14^ This therapy is designed to train strategies that support lexical retrieval by strengthening the patient’s remaining semantic, orthographic and phonological knowledge. LRT has shown efficacy in people with PPA even in the absence of neuromodulation. The treatment followed a structured series of tasks aimed at activating and reinforcing the different components of language processing. It began with techniques based on semantic self-cueing and progressively introduced orthographic and phonemic cues to help facilitate word finding.

In each session, five words were trained. Each word was practiced through the structured sequence of steps lasting ∼10 min. The word selection was guided by a preliminary naming task involving a list of 261 items. Only words that were not spontaneously named correctly were chosen for training. There was no predefined minimum or maximum number of items to be trained across the intervention period. Instead, therapy targeted items that were eligible at each assessment time point. The same item selection procedure and therapy protocol were applied across all PPA variants. The steps followed to practice each selected word are detailed in Supplementary Table 3.

Although LRT is primarily designed to address naming deficits, anomia is commonly present across all PPA variants, including nfvPPA. Moreover, LRT targets multiple components of language processing, including semantic, phonological and orthographic systems, as well as speech production and sentence construction. Therefore, we decided to apply the same language intervention across all PPA variants, given its potential benefits for the anomia associated with the three variants and beyond naming.

### Clinical endpoint variables

#### Mini Linguistic State Examination

The Spanish version of the MLSE^15^ was administered to participants at baseline, after 3 months and at the end of the treatment. The MLSE was designed to be a brief but comprehensive test of language ability, intended to be used in the assessment, clinical classification and follow-up of progressive aphasic syndromes. It consists of 11 tasks evaluating key language domains typically affected in PPA.^16^ The test takes an average of 20 min to administer. These tasks are structured to assess motor speech, phonology, semantics, syntax and verbal working memory. The test score counts the number of errors that correspond to each domain, providing a score profile across five dimensions. A total score out of 100 can also be computed to reflect overall language performance.

#### Words per minute

WPM was used as a quantitative measure of spontaneous speech production. Participants were asked to tell a story based on scenes from wordless picture books over a period of 3 min, and their responses were recorded and transcribed. All the words spoken by the patient were counted and divided by the total seconds of the recording. The result was multiplied by 60 s to obtain the number of words per minute. This task was administered to participants at baseline, after 3 months and at the end of the treatment.

#### Confrontation naming list

The confrontation naming task consisted of a list of 261 words, encompassing items from eight semantic categories: animals, food, clothing, body parts, transport, objects, furniture and places. These words were selected based on prior clinical experience with lexical retrieval training in this population, including words with varying frequency of use that would be meaningful and applicable to everyday life in this population. This task was designed to evaluate naming ability. Participants were asked to name visually presented items, and performance was assessed based on the total number of correctly named items without any provided cues. The list of words was administered to participants at baseline, after 3 months and at the end of the treatment. The full list of items is provided in Supplementary Table 4.

#### Interview for Deterioration in Daily Living Activities in Dementia

The IDDD is a caregiver-reported questionnaire developed to assess functional decline in everyday activities associated with cognitive impairment. It consists of 33 items divided into two subscales: one measuring basic activities of daily living (e.g. dressing) and the other assessing instrumental activities of daily living (e.g. using the telephone). Higher scores reflect greater levels of functional deterioration.^17^ The Spanish version of the IDDD^18^ was completed by patients’ relatives or caregivers at baseline, after 3 months and at the end of the treatment period.

#### Neuropsychiatric Inventory

The NPI is a structured interview administered to caregivers to evaluate neuropsychiatric symptoms commonly associated with neurodegenerative disorders.^19^ It covers 12 domains such as delusions, hallucinations, agitation/aggression, dysphoria/depression, anxiety, euphoria/elation, apathy/indifference, disinhibition, irritability/lability, aberrant motor behaviours, night-time behavioural disturbances and appetite/eating disturbances. Each symptom is rated for frequency and severity, yielding a total score that reflects the overall neuropsychiatric symptoms. Higher scores indicate more severe neuropsychiatric symptoms on the NPI. The Spanish version of the NPI^20^ was completed by the patients’ relatives or caregivers at the beginning of the treatment, 3 months later and at the end of the treatment.

### Baseline clinical and cognitive assessments

Clinical and demographic information was obtained through interviews with the patients or their relatives. Collected data included age, sex and educational background.

#### Cognitive assessment

##### Addenbrooke’s Cognitive Examination III

All participants completed the Spanish version of the Addenbrooke’s Cognitive Examination III (ACE-III).^21^ This tool is broadly employed in clinical settings to screen for cognitive impairment and is particularly useful in the detection of dementia and other neurocognitive disorders, including Alzheimer’s disease, frontotemporal dementia and primary progressive aphasia.^22-25^ It assesses five major cognitive domains: attention and orientation (/18), memory (/26), verbal fluency (/14), language (/26) and visuospatial skills (/16). The total score ranges from 0 to 100, with higher scores reflecting better global cognitive functioning.

##### Neuronorma battery

All participants completed a selection of tests from the Neuronorma battery,^26^ a comprehensive set of neuropsychological tests. This battery includes instruments aimed at evaluating attention, episodic memory, executive functioning and visuospatial abilities.

The tests administered included Digit Span (forward and backward), Corsi Block Tapping Test (forward and backward), Trail Making Test (Part A & B), Symbol Digit Modalities Test, subtests from the Visual Object and Space Perception Battery (VOSP), Judgment of Line Orientation, Tower of London, Rey–Osterrieth Complex Figure Test (copy, 3-min recall, 30-min recall and recognition) (ROCF) and the Stroop Test.

##### Language assessment

A comprehensive set of language assessments was administered to all participants. This battery encompassed a range of tasks targeting key language domains such as reading, comprehension, repetition, spontaneous speech, grammatical processing, naming and semantic memory.

The specific tasks included reading of single words, capitalized words, foreign words and non-words; initial phoneme omission; word spelling; repetition of words, non-words, syllables, pairs of syllables and sentences; semantic association; picture and action naming; word-picture and picture-action matching; synonym judgment; verb tense agreement; and complex sentence comprehension.

A more detailed description of each task used can be found in Supplementary Table 5 and elsewhere.^27,28^

### FDG-PET acquisition, preprocessing and data extraction

All participants were examined using ^18^F-FDG-PET. Images were acquired in a Siemens Biograph TruePoint PET-CT scanner. Patients fasted for at least 6 h prior to the scan. The dose was administered 30 min before image acquisition. Current European recommendations for the acquisition of FDG-PET brain studies were followed.^29^ Images were processed using Statistical Parametric Mapping version 12. Images were realigned to the AC/PC plane and normalized to the Montreal Neurological Institute space using a template previously validated for neurodegenerative disorders.^30^ Marsbar software (https://marsbar-toolbox.github.io/download.html) was used for region of interest analysis, including all the regions of the Automated Anatomical Labeling (AAL) atlas, and adjusting by the global mean intensity. All regions are specified in Supplementary Table 6.

### Statistical analysis

Statistical analyses were conducted using RStudio version 2024.04.2+764. Descriptive results were shown as frequency (percentage) and mean ± standard deviation. We calculated the correlations between variables at baseline and the change in each variable using Pearson’s correlation coefficient. The magnitude of the correlation was interpreted according to the following criteria: small (*r* < 0.30), moderate (0.30 ≤ *r* < 0.50) and large (*r* > 0.50).

To identify predictors of clinical change in language abilities, neuropsychiatric symptoms and functional outcomes, we used least absolute shrinkage and selection operator (LASSO) regression to identify the most relevant predictors at 6 months. LASSO is a machine-learning technique that performs both variable selection and regularization, shrinking the coefficients of less informative variables to zero, thereby enhancing model interpretability and reducing the risk of overfitting. For each clinical endpoint, we calculated the 6-month change score (e.g. MLSE at 6 months minus MLSE at baseline). Predictor variables included all baseline demographic, clinical and neuropsychological scores and PET uptake in AAL regions. Treatment condition (e.g. active TMS or sham) was also included as a predictor. Predictors were standardized and entered into the model using a design matrix generated by model.matrix(). We used 5-fold cross-validation to identify the optimal regularization parameter (lambda) that minimized the cross-validated mean squared error. Models were fitted using the glmnet package with alpha = 1 to enforce the L1 penalty (LASSO). Final models included only variables with non-zero coefficients at the optimal lambda.

To further interrogate the predictors of clinical change (i.e. language, neuropsychiatric and functional), Elastic Net regression was also used. This approach combines the strengths of both Ridge and LASSO regularization methods by incorporating both L1 and L2 penalties, which helps to overcome some of the limitations of LASSO regression when there are highly correlated predictors. The Elastic Net model was fitted using the glmnet package in R. The optimal penalty parameter (λ) was selected via 5-fold cross-validation. Specifically, the cv.glmnet() function was used to perform cross-validation, minimizing the cross-validated mean squared error (MSE) to select the best λ. The resulting model coefficients were estimated using the glmnet() function with the chosen λ.

Model performance was evaluated using *R*-squared (*R*^2^) and root mean squared error (RMSE). To avoid overinterpreting poorly performing models, only those with a cross-validated *R*^2^ of 0.30 or higher were retained in the main results. In order to highlight the most influential predictors, only variables with absolute coefficients greater than 0.1 were specified in the text and Fig. 1. Models with lower performance were considered exploratory and included in Supplementary Tables 7–11.

**Figure 1:**
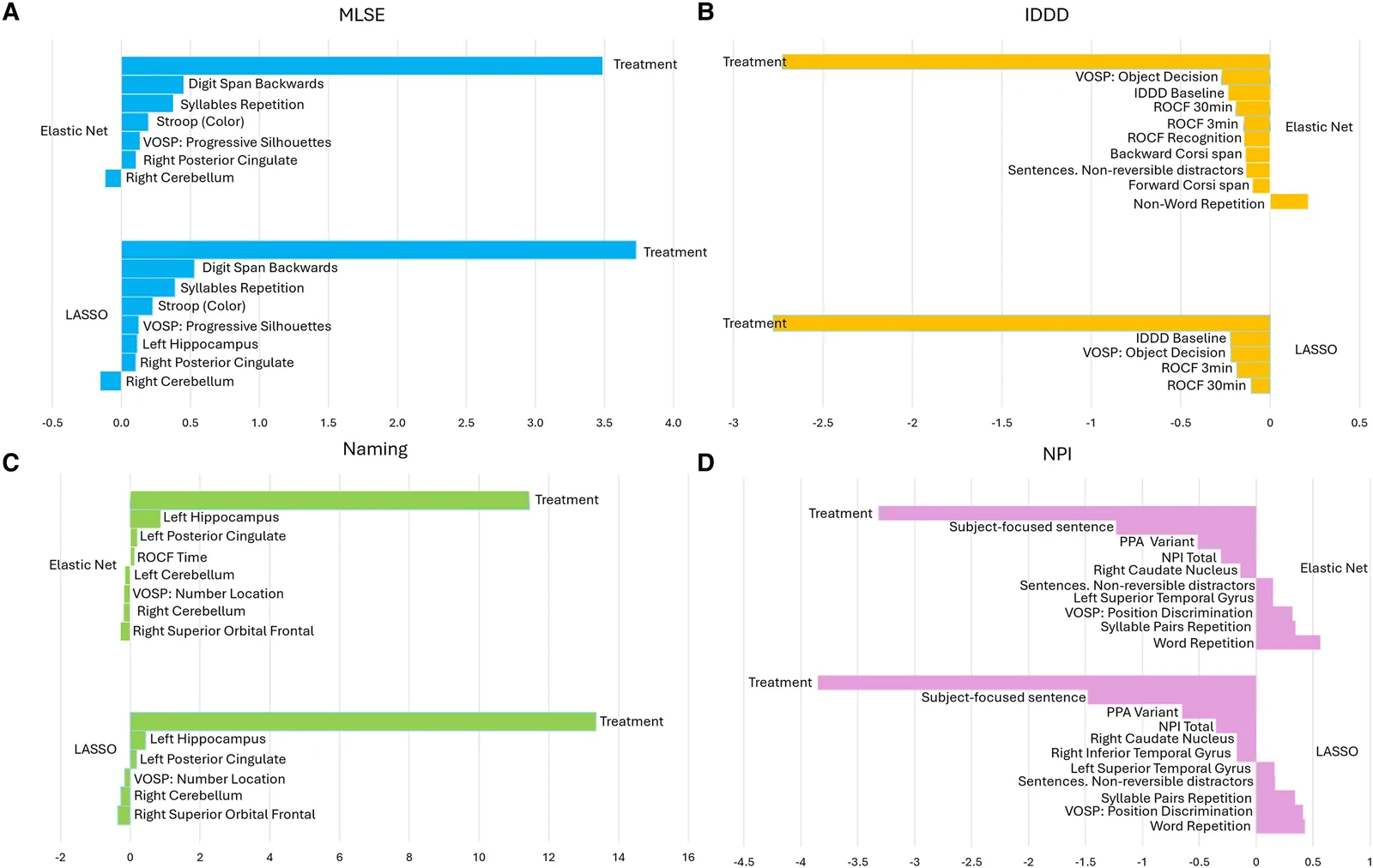
**Predictors of clinical change in PPA according to LASSO and Elastic Net regression models.** Bar plots represent standardized coefficients (absolute value >0.1) from the best-performing models (*R*^2^ ≥ 0.30) for each clinical outcome: (**A**) MLSE, (**B**) IDDD, (**C**) naming and (**D**) NPI. Abbreviations: MLSE, Mini Linguistic State Examination; IDDD, Interview for Deterioration in Daily Living in Dementia; NPI, Neuropsychiatric Inventory.

To identify factors associated with response to TMS therapy, we performed an unsupervised clustering analysis using k-means to identify distinct subgroups of patients based on their clinical change (clinical variables at 6 months minus baseline). The analysis focused on quantifying overall patterns of change over the 6-month intervention period on the clinical variables: MLSE, WPM, naming, IDDD and NPI. Before clustering, all change score variables were standardized (z-scores) to ensure equal weighting across different scales. The optimal number of clusters was determined using the elbow method, which examines the within-cluster sum of squares for increasing numbers of clusters. We used 25 random starts and a maximum of 100 iterations. After clustering, we examined the characteristics of the clusters, and we compared the clinical and cognitive characteristics specifically in the group of patients receiving active TMS to identify those characteristics associated with each cluster. Non-parametric group comparisons were performed using the Mann–Whitney U test.

## Results

### Correlations between clinical variables

Correlations between the change of the clinical variables from baseline to 6-month assessments were moderate yet significant between MLSE and naming (*r* = 0.379, *P* = 0.004), MLSE and IDDD (*r* = –0.359, *P* = 0.007), and naming and IDDD (*r* = –0.440, *P* = 0.001). The other correlations were not statistically significant. All correlations are shown in Supplementary Table 12.

### Prediction of MLSE change

The LASSO model showed good performance, with an *R*^2^ of 0.52 and an RMSE of 4.94. The variables retained included treatment with TMS, digit span backwards, Stroop colour naming, VOSP (progressive silhouettes), repetition of syllables and brain metabolism in the right cerebellum, the right posterior cingulate and the left hippocampus.

The Elastic Net model achieved an *R*^2^ of 0.57 and an RMSE of 5.05. The variables selected included treatment with TMS, digit span backwards, Stroop colour naming, VOSP (progressive silhouettes), repetition of syllables and brain metabolism in the right cerebellum and right posterior cingulate.

### Prediction of naming change

The LASSO regression model yielded an *R*^2^ = 0.422 and an RMSE of 15.0. The variables retained included treatment with TMS, VOSP (number location) and brain metabolism in the right cerebellum, left posterior cingulate, right superior orbital frontal cortex and left hippocampus.

The predictive model built using Elastic Net regularization yielded an *R*^2^ of 0.41 and an RMSE of 15.17. The predictors identified included treatment with TMS, ROCF (time), VOSP (number location) and brain metabolism in the left cerebellum, right cerebellum, left posterior cingulate, right superior orbital frontal cortex and left hippocampus.

### Prediction of WPM change

In this case, both LASSO regression and Elastic Net models showed modest or no meaningful predictive capacity. The LASSO model yielded an *R*^2^ of 0.19 and RMSE of 16.7. Elastic Net achieved *R*^2^ = 0.19 and RMSE of 16.65. The predictors identified in the LASSO regression model included sentence comprehension (passive sentences) and brain metabolism in the left superior occipital gyrus. The predictors identified through Elastic Net were sentence comprehension (passive sentences) and brain metabolism in the right amygdala and the left superior occipital gyrus.

### Prediction of IDDD change

Model performance was *R*^2^ = 0.462 and RMSE = 6.12 using LASSO regression. The variables retained included treatment with TMS, IDDD score, ROCF (3 min recall and 30 min recall) and VOSP (object decision).

The Elastic Net model provided an *R*^2^ of 0.58 and an RMSE of 5.35. The predictor variables retained in the model were treatment with TMS, IDDD, Corsi span forward and backwards, ROCF (3 min recall, 30 min recall and recognition), VOSP (object decision), sentence comprehension (non-reversible distractors) and repetition of non-words.

### Prediction of NPI change

The LASSO regression model yielded an *R*^2^ of 0.807 and an RMSE of 3.47. The predictors identified included treatment with TMS, PPA variant, NPI total score, VOSP (position discrimination), sentence comprehension (subject-focused sentence and non-reversible distractors), repetition of pairs of syllables and words, and brain metabolism in the right caudate nucleus, right inferior temporal gyrus and left superior temporal gyrus.

Using Elastic Net regularization, the *R*^2^ was 0.769 with an RMSE of 3.79. The variables retained included treatment with TMS, PPA variant, NPI total score, VOSP (position discrimination), sentence comprehension (subject-focused sentence and non-reversible distractors), repetition of pairs of syllables and words, and brain metabolism in the right caudate nucleus and left superior temporal gyrus.

### K-means clustering

The optimal clustering solution was *k* = 2, yielding two distinct groups: a non-responder group (Cluster 1, *n* = 23) and a responder group (Cluster 2, *n* = 34). A significantly higher proportion of patients who received active TMS were classified into the responder group (Cluster 2: 31/40, 77.5%; Cluster 1: 9/40, 22.5%), while the majority of patients who received sham TMS were part of the non-responder group (Cluster 1: 14/17, 82.4%; Cluster 2: 3/17, 17.6%) (χ^2^ = 17.75, *P* < 0.001) (Fig. 2). No significant differences were observed in the distribution of PPA variants across clusters (χ^2^ = 2.50, *P* = 0.284).

**Figure 2:**
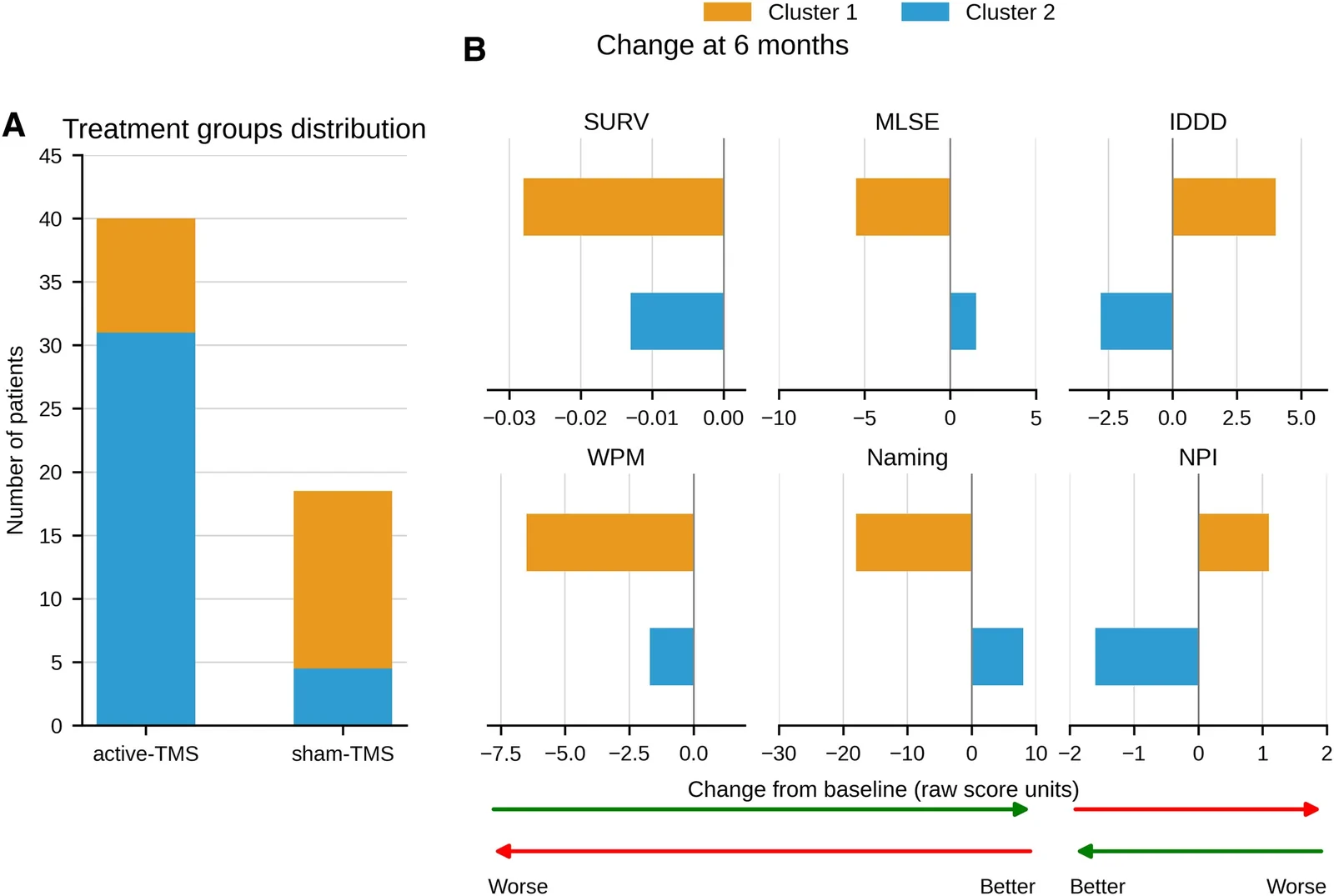
**Treatment group distribution and 6-month clinical changes according to response cluster.** (A) Proportion of participants classified as responders (Cluster 2, lower segment of each bar) or non-responders (Cluster 1, upper segment) within the active and sham TMS groups. Differences in treatment group distribution between clusters were assessed using Pearson’s χ² test (χ² = 17.75, p < 0.001). (B) Change scores from baseline to 6 months in outcome measures. In each plot, the upper bar corresponds to Cluster 1 (non-responders) and the lower bar to Cluster 2 (responders). Arrows below the plots indicate the direction of clinical improvement (“Better”) and worsening (“Worse”): for SUVR, MLSE, WPM and Naming, positive values indicate improvement, whereas for IDDD and NPI, negative values indicate improvement. Abbreviations: TMS = transcranial magnetic stimulation; SUVR = standardised uptake value ratio; MLSE = Mini Linguistic State Examination; WPM = words per minute; IDDD = Interview for Deterioration in Daily Living Activities in Dementia; NPI = Neuropsychiatric Inventory.

We then examined the clinical and demographic characteristics of patients who received active TMS, stratified by their membership in Cluster 1 (non-responders) or Cluster 2 (responders). There were differences in the distribution of PPA variants between clusters (χ^2^ = 6.35, *P* = 0.042). Specifically, Cluster 1 (non-responders) included 2 patients with nfvPPA, 0 with svPPA and 7 with lvPPA, whereas Cluster 2 (responders) comprised 14 patients with nfvPPA, 7 with svPPA and 10 with lvPPA. At baseline, patients in Cluster 1 showed lower scores in the ACE-III memory domain, ACE-III visuospatial domain and the ROCF delayed recall (3-min) task (Fig. 3). There were no other significant differences in demographic, cognitive or language characteristics (Supplementary Table 13). Cluster 1 showed lower metabolism in the left angular and supramarginal gyri, bilateral Heschl’s gyri, left hippocampus, left inferior parietal lobule, and left inferior and middle temporal gyri, as well as higher cerebellar and right supplementary motor area metabolism compared to Cluster 2 (Fig. 4).

**Figure 3:**
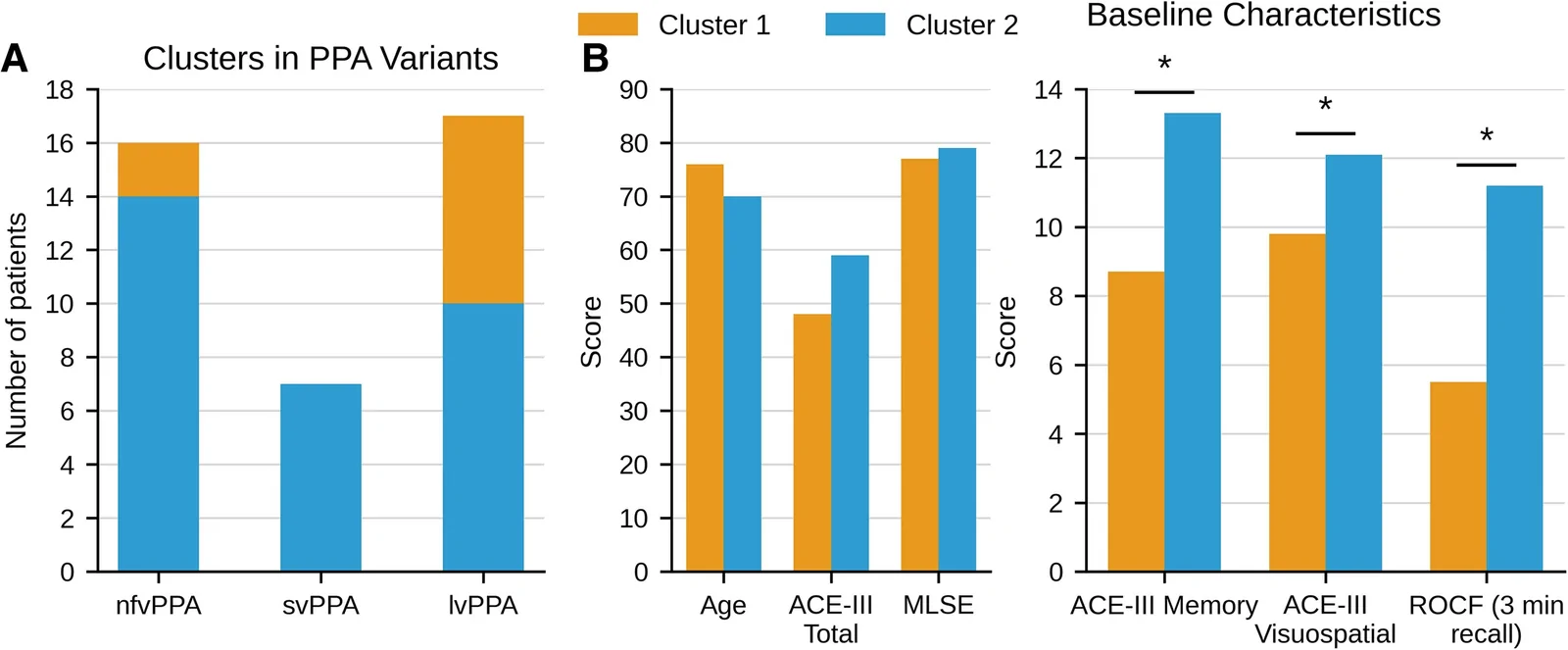
**Characteristics of the active TMS group by clusters.** (A) Distribution of PPA variants by cluster. In each stacked bar, the lower segment corresponds to Cluster 2 (responders) and the upper segment to Cluster 1 (non-responders). Differences in PPA variant distribution between clusters were assessed using Pearson’s χ² test (χ² = 6.35, p = 0.042). B) Baseline characteristics by cluster. For each variable, the left bar corresponds to Cluster 1 (non-responders) and the right bar to Cluster 2 (responders). Baseline comparisons were performed using the Mann–Whitney U test. Significant differences are indicated by * (p < 0.05). Abbreviations: PPA = Primary Progressive Aphasia; nfv = non-fluent variant; sv = semantic variant; lv = logopenic variant; ACE-III = Addenbrooke’s Cognitive Examination III; MLSE = Mini Linguistic State Examination; ROCF = Rey–Osterrieth Complex Figure.

**Figure 4:**
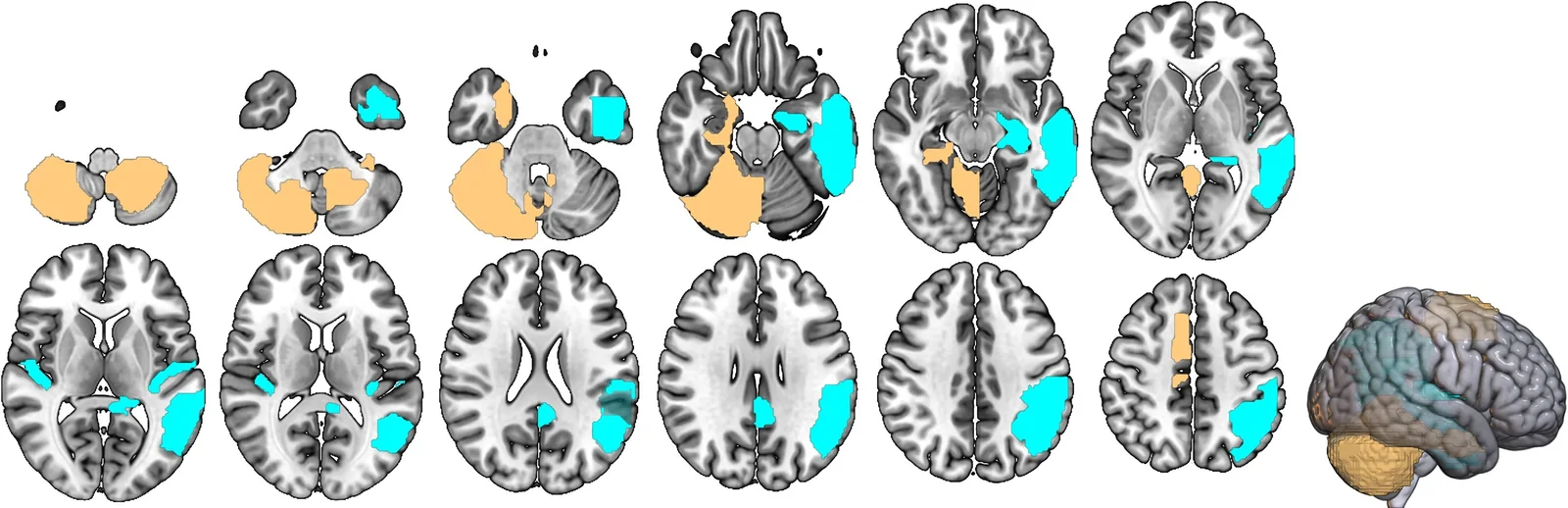
**Brain regions associated with clinical response.** Brain regions associated with clinical response. Higher brain metabolism in the cerebellum (bilaterally) and the right superior orbitofrontal cortex was associated with poorer treatment response. Lower baseline metabolism in the left lateral temporal, temporoparietal and posterior cingulate/precuneus regions was associated with poorer treatment response.

## Discussion

In this study, we evaluated the ability of various demographic, clinical and FDG-PET imaging measures to predict clinical change at 6 months in patients with PPA. All participants were enrolled in a clinical trial in which they received evidence-based language therapy, along with either active or sham TMS in a 2:1 ratio. The models for MLSE, naming, IDDD and NPI showed moderate to good fit according to *R*^2^ and good to excellent precision following RMSE.

One of the most striking findings of our study is the identification of the different variables most predictive of clinical change. In addition, our results suggest that response to language therapy in PPA is not uniform and is influenced by a combination of clinical, cognitive and neurobiological factors. The fact that TMS treatment emerged as the strongest predictor of all clinical variables reported herein (MLSE, naming, IDDD, NPI) underscores its promise as a therapeutic intervention in PPA, supporting the value of this technique to (i) maximize the effects of evidence-based language training and (ii) predict clinical change (i.e. language, functional and neuropsychiatric progression). Specifically, active TMS was predictive of clinical improvement. It is worth mentioning that, in this study, TMS was targeted to the left DLPFC. The selection of the DLPFC as the stimulation target was based on its role as a key hub within frontoparietal control networks involved in executive and language-related processes,^31^ as well as its widespread connectivity with temporal and parietal regions supporting language function.^32,33^ In addition, the DLPFC is typically relatively preserved in the early stages of PPA compared to core language regions, making it a suitable target to modulate distributed networks without directly stimulating areas that are more severely affected (and potentially with a low response to brain stimulation). This network-based approach may help explain the observed therapeutic effects of TMS in our study. It is also noteworthy that the PPA variant was only predictive of change on the neuropsychiatric scale. On one hand, this aligns with previous findings, as certain PPA subtypes (particularly the non-fluent/agrammatic and semantic variants) are known to exhibit more prominent behavioural and emotional changes over time.^34^ On the other hand, the lack of predictive value of PPA variant for the other outcome measures may reflect the broader and more balanced nature of these instruments, with scores representing changes across several language domains (e.g. MLSE) or functions affected across the three variants (naming, functional impairment).^16,35,36^

Furthermore, we found that baseline general cognitive performance was a significant predictor of clinical change, probably allowing patients to better engage with language therapy. In this regard, higher performance on some attention and working memory tasks were predictive of improvement on the MLSE, and lower performance on episodic memory, attention and visuospatial tests were related to greater functional impairment over time. It was unexpected that variables related to language performance were not identified as important predictors in the models predicting clinical change, even though we added a good number of variables covering the main language functions as predictors to the statistical models. This might suggest that the severity of language impairment may not predict therapeutic success, indicating that the language therapy used in our study should not be withheld from patients with more severe language deficits. However, it is important to note that all patients included in the clinical trial were in relatively early stages of PPA and were able to engage in the therapy. Visual inspection of baseline score distributions did not indicate relevant ceiling or floor effects for the language measures, which showed adequate variability across participants. Therefore, future studies specifically designed to assess the effectiveness of the intervention across different disease stages are needed.^37^ This finding may partly reflect a restricted range of baseline language impairment due to the clinical trial inclusion criteria, which may have limited variability and reduced the ability of baseline scores to predict subsequent change. In addition, treatment response in PPA may not be linearly related to baseline severity. Instead, it may depend more strongly on other factors, such as neuroanatomical patterns of degeneration, the integrity of preserved brain networks or cognitive reserve, which are not fully captured by baseline language measures alone. Finally, the use of penalized regression methods may have further reduced the likelihood of selecting baseline language variables, particularly when correlated predictors accounted for a greater proportion of variance in the outcome.

Regarding behaviour, the models showed good predictive performance, once again highlighting the positive effect of TMS treatment, which supports the use of this intervention in symptoms such as apathy.^38^ Additionally, associations were observed with various cognitive functions (both linguistic and non-linguistic) as well as with cortical and subcortical regions, consistent with the widespread structural and functional bases of neuropsychiatric disorders.^39^

Unfortunately, the model for WPM change showed poor performance. This is consistent with the high variability of this measure detected in the clinical trial,^11^ which suggests the need to examine other measures to reliably capture spontaneous speech in patients with PPA.^40^ The lack of correlation with the other language outcomes also confirms that other variables would prove useful in the evaluation of connected speech in future studies. It is important to note that WPM reflects a combination of speech production and language processes. As pauses were not explicitly accounted for, this measure may also capture word-finding difficulties in addition to speech production itself. It is also possible that the particular connected speech features associated with treatment response may vary as a function of treatment type and clinical variant, which should be investigated in future studies.

In the unsupervised clustering analysis, two subgroups of patients were identified according to the changes at 6-month follow-up in language, functional and behavioural outcomes. A large proportion of patients receiving active TMS were classified as responders, whereas most of the patients in the sham group were considered non-responders. Interestingly, all participants in the active TMS condition with svPPA and 87.5% of those with nfvPPA were classified as responders. The relatively high response observed in nfvPPA may appear somewhat surprising, given that naming deficits are not typically the core impairment in this variant. Nevertheless, all participants in our sample presented with anomia at baseline, and the language therapy targeted not only naming but also sentence construction, semantic elaboration and speech production, which may explain the observed response. Future studies specifically focused on patients with isolated speech disorders (e.g. progressive apraxia of speech) would be useful to better characterize the response to this type of intervention. Regarding patients with lvPPA, the percentage of responders was lower (58.8%). This finding differs from previous reports showing comparable treatment responses between svPPA and lvPPA.^12^ One possible explanation is that, in our study, new lexical items were introduced progressively throughout the 6-month intervention. As a result, items trained later may have been more susceptible to disease progression before treatment, potentially contributing to the lower proportion of responders observed in participants with lvPPA.^24^ Additionally, the TMS target used in our study (DLPFC) may be less optimal for this variant, given the pattern of cortical atrophy typically observed in lvPPA. In addition, lower scores in non-language domains (memory and visuospatial) were associated with a suboptimal treatment response. This could also be interpreted in light of the possibility that cognitive deficits may limit the effectiveness of combined TMS and language therapy, as has been suggested for spelling therapy during transcranial direct current stimulation.^41^ Alternatively, in cases with more pronounced memory impairments, other TMS targets (e.g. precuneus) could be more appropriate instead of DLPFC, as has been proposed in amnestic Alzheimer’s disease.^42^ Future studies should investigate whether individualized stimulation targets based on neuroimaging findings and clinical phenotype improve treatment response across PPA variants.

Regarding the effect on naming, it should also be noted that the present study was not designed to differentiate between trained and untrained items, and a separate set of untrained items was not defined. While this approach reflects the clinical implementation of the therapy, it may have limited the ability to detect variant-specific effects and could partly account for differences between our findings and those reported in previous studies.^12^

Interestingly, patients with nfvPPA showed a relatively strong response to treatment, which may appear unexpected given that naming deficits are not typically the core impairment in this variant. However, all participants in our sample presented with anomia at baseline. In addition, the therapy used did not exclusively target naming but also included sentence construction, semantic elaboration and speech production.

Our study also identified several brain regions that were predictive of clinical change. Notably, areas such as the left hippocampus and the left posterior cingulate were associated with language improvement. This finding is consistent with previous work showing that hippocampal integrity may predict language treatment response in PPA, particularly in svPPA.^43^ In general, higher metabolism in these regions predicted more favourable clinical outcomes, suggesting that the integrity of certain key brain areas may be important for therapeutic success. Interestingly, the regions identified were not those typically most affected in PPA. In this regard, we also included a region of interest encompassing all areas commonly impaired in PPA, but this composite region did not predict clinical change. Overall, these findings suggest that the severity of language dysfunction or damage to core language regions may not be the primary driver of clinical change. Instead, clinical change may be more strongly related to the degree of preservation or disruption to brain areas supporting non-language functions. However, given the topographical heterogeneity across PPA variants, future studies focusing on variant-specific predictive patterns are necessary.

Interestingly, cerebellar hypermetabolism was associated with non-responders. One possible explanation for this finding is that it could reflect a compensatory mechanism in response to cortical dysfunction which, instead of supporting recovery, could be maladaptive, as has been suggested by some authors.^44^ Notably, the cerebellum is functionally integrated, maintaining widespread connections with the cerebral cortex, including neocortical regions involved in higher-order cognitive processes. Cerebellar hyperactivity may interfere with synaptic plasticity and disrupt the functional balance of cerebro-cerebellar circuits, thereby limiting the efficacy of TMS and language therapy. Recent evidence has highlighted its role in the pathophysiology of neurodegenerative diseases such as frontotemporal dementia, PPA, and Alzheimer’s disease.^45^ To this end, some authors have proposed the cerebellum as a potential target for brain stimulation in PPA.^45-47^

Our study has some limitations. First, we performed LASSO regression, which produces simpler, more interpretable results because of the variable selection procedure. LASSO is especially useful in datasets with many variables compared to the sample size (*P* > *n*), because it performs automatic variable selection and minimizes the risk of overfitting. In addition, the use of cross-validation to determine the optimal lambda adds robustness to the model selection process. Feature selection in LASSO may be limited if there are several correlated predictors. For this reason, we also implemented Elastic Net, which largely confirmed the robustness of the models and the predictors identified with LASSO. Although penalized regression and cross-validation were used to reduce overfitting, the relatively small sample size compared to the number of predictors warrants cautious interpretation, and further studies are needed to validate these predictors in independent samples. Due to the limited sample size, we did not include treatment as an interaction term in the regression model to evaluate specific predictors of response to TMS, as this could compromise model performance by increasing the risk of overfitting and reducing generalizability. Instead, we applied unsupervised clustering, which identified two subgroups based on clinical change. This approach allowed us to explore factors associated with response to TMS. Second, we did not formally assess the integrity of masking. Although informal feedback from caregivers suggested that participants were generally unable to distinguish between active and sham conditions, future studies should formally evaluate this. Third, we did not examine changes in individual neuropsychiatric symptoms, which may have limited the identification of domain-specific treatment effects. In addition, the intervention protocol requires substantial commitment from both patients and caregivers, particularly regarding transportation and time demands, and one participant discontinued the study at 3 months due to caregiver difficulties in attending hospital visits. As our study was conducted in an urban setting with good access to hospital resources, these factors may limit generalizability to less accessible contexts. Future studies should explore more scalable approaches, including home-based neuromodulation techniques.

## Conclusions

In conclusion, we developed predictive models using penalized regressions to estimate clinical changes at 6 months in patients with PPA following evidence-based language therapy. Our findings suggest that response to language and brain stimulation therapy may not be uniform. Notably, TMS treatment emerged as the primary variable associated with clinical improvement, underscoring the benefits that extend beyond treatment response. Furthermore, better cognitive performance and more preserved brain metabolism in several key regions were associated with a more favourable clinical trajectory, indicating a potential responder profile for language therapy. Accurate prediction of clinical change in PPA holds implications for patients and families and also for the design of emerging clinical trials. Tailoring interventions based on predictive response profiles may ultimately enhance treatment efficacy and move the field towards precision medicine approaches to care.

## Supplementary Material

fcag398_Supplementary_Data

## Data Availability

The dataset is available from the corresponding author upon reasonable request.

## Study registration

This study is a subanalysis of a clinical trial pre-registered (ClinicalTrials.gov identifier: NCT05842473)
