# Supplementary material for: Predictors of 6-month clinical change in primary progressive aphasia following language therapy and transcranial magnetic stimulation
Source: Brain Commun. 2026 Oct 19;8(6):fcag398. doi: 10.1093/braincomms/fcag398 (PMC13725162; doi:10.1093/braincomms/fcag398)
Supplement: Supplementary file 1 [file fcag398_supplementary_data.docx]

**SUPPLEMENTARY MATERIAL**

**Supplementary Table 1.** Patient Inclusion and Exclusion Criteria

**Supplementary Table 2.** Baseline demographic and clinical characteristics (per protocol analysis for the primary outcome)

**Supplementary Table 3.** Summary of Treatment Steps Based on Lexical Retrieval Treatment (Language Intervention)

**Supplementary Table 4.** Registration of the confrontation naming list

**Supplementary Table 5**. Summary of Cognitive and Language Assessment Tests in PPA

**Supplementary Table 6.** Brain Metabolism Differences Between Clusters

**Supplementary Table 7**. Coefficients of LASSO and Elastic Net analysis for MLSE

**Supplementary Table 8.** Coefficients of LASSO and Elastic Net analysis for Naming

**Supplementary Table 9**. Coefficients of LASSO and Elastic Net analysis for IDDD

**Supplementary Table 10.** Coefficients of LASSO and Elastic Net analysis for NPI

**Supplementary Table 11.** Coefficients of LASSO and Elastic Net analysis for WPM

**Supplementary Table 12.** Correlations between clinical variables

**Supplementary Table 13.** Clinical and Demographic Characteristics of the Active TMS Group Across Clusters at Baseline.

**Supplementary Figure 1.** Distribution of baseline outcome measures

**Supplementary Table 1.** Patient Inclusion and Exclusion Criteria

| Inclusion criteria | Exclusion criteria |
| --- | --- |
| Diagnosis of primary progressive aphasia (PPA) according to Gorno-Tempini et al., supported by FDG-PET and/or MRI | Diagnosis of a neurological or medical condition other than PPA that could explain language impairment |
| Clinical Dementia Rating (CDR) score ≤ 1 | History of epilepsy or epileptiform activity on EEG |
| Language impairment as the primary clinical deficit | Contraindications to TMS or MRI (e.g., ferromagnetic implants, intracranial devices, metallic fragments, or pregnancy) |
| Written informed consent provided by the patient or legal representative | Pregnancy, breastfeeding, or plans to become pregnant (contraception required when applicable) |
|  | Terminal illness with life expectancy <1 year |
|  | Active malignancy within the past 2 years |
|  | Any condition preventing participation or follow-up |
|  | Alcohol or substance abuse within the past year |
|  | Major psychiatric disorders (e.g., schizophrenia, bipolar disorder, OCD, personality disorders) |
|  | Inability to communicate (e.g., mutism) or insufficient language proficiency |
|  | Severity of PPA preventing participation in study procedures |
|  | Participation in another clinical trial within the past 4 months |
|  | Use of medications affecting study outcomes unless stable ≥3 months (e.g., antiepileptics, benzodiazepines, cholinesterase inhibitors, memantine, SSRIs, seizure-threshold–lowering drugs) |

| **Supplementary Table 2.** Baseline demographic and clinical characteristics (per protocol analysis for the primary outcome) | | | | |
| --- | --- | --- | --- | --- |
|  | | Active group  (n=40) | Sham group  (n=17) | U (p-value) |
| *Demographic* | | | | |
| Age, mean (SD), years | | 71.52 (8.33) | 71.33 (9.02) | 346 (0.814) |
| Sex, Females | | 30 (75.0%) | 8 (44.4%) | 5.13 (0.024) |
| Race, White | | 40 (100%) | 18 (100%) | - |
| Education, years | | 13.55 (5.10) | 13.72 (4.04) | 358 (0.979) |
| *Clinical* | | | | |
| Time since symptom onset, months | | 29.33 (21.97) | 27.87 (17.305) | 3.121 (0.786) |
| PPA variant | nfvPPA | 16 (40.0%) | 6 (35.3%) | 0.306 (0.858) |
|  | svPPA | 7 (17.5%) | 3 (17.6%) |  |
|  | lvPPA | 17 (42.5%) | 8 (47.1%) |  |
| ACE-III (total score) | | 55.92 (18.85) | 42.28 (21.63) | 258 (0.086) |
| MLSE | | 79.13 (11.39) | 75.39 (14.82) | 318 (0.485) |
| NPI | | 9.03 (12.01) | 8.39 (13.34) | 308 (0.384) |
| IDDD | | 47.32 (13.34) | 45.50 (11.05) | 358 (0.980) |

Note: Values are presented as mean (SD), unless otherwise indicated. Abbreviations: ACE: Addenbrooke’s Cognitive Examination; IDDD: Interview for Deterioration of Daily Living in Dementia; MLSE: Mini-Linguistic State Examination; NPI: Neuropsychiatric Inventory; PPA: primary progressive aphasia.

**Supplementary Table 3.** Summary of Treatment Steps Based on Lexical Retrieval Treatment (Language Intervention)

| Semantic Feature Analysis | - Prompt semantic description of object with “Tell me about it.” - Clinician then prompts with follow-up questions to elicit important semantic features associated with the object. - Clinician provides summary of participant’s description - Participant asked to recall name of object. |
| --- | --- |
| Orthographic /Phonemic Self-Cue | - Clinician prompts written production of the target word, “Can you write the word?” - If unable to do so, clinician prompts for initial phoneme “Can you say the first sound?” |
| Written and spoken repetition | - Clinician prompts participant to copy of and read written word three times |
| Semantic plausibility judgments | - Clinician asks participant yes/no questions regarding semantic features of target (x5) |
| Recall | - Clinician asks participant to recall two important semantic features and to write and say the name from memory once |
| Description of a complex picture | - Clinician provides a complex image with the target included in the scene and asks participant to describe the picture using the target word |

**Supplementary Table 4.** Registration of the confrontation naming list

Gato (Cat)
Perro (Dog)
Vaca (Cow)
Cerdo (Pig)
Gallina (Chicken)
Mono (Monkey)
Cebra (Zebra)
Pez (Fish)
Serpiente (Snake)
Cabra (Goat)
Elefante (Elephant)
León (Lion)
Tortuga (Turtle)
Tigre (Tiger)
Gusano/Lombriz (Worm)
Delfín (Dolphin)
Caracol (Snail)
Loro (Parrot)
Zorro (Fox)
Burro (Donkey)
Caballo (Horse)
Águila (Eagle)
Hipopótamo (Hippopotamus)
Pingüino (Penguin)
Pato (Duck)
Cocodrilo (Crocodile)
Rata/Ratón (Rat/Mouse)
Mariposa (Butterfly)
Búho (Owl)
Abeja (Bee)
Ardilla (Squirrel)
Jirafa (Giraffe)
Oso (Bear)
Foca (Seal)
Tiburón (Shark)
Rinoceronte (Rhinoceros)
Oveja (Sheep)
Camello (Camel)
Rana (Frog)

Avión (Airplane)
Coche (Car)
Bici (Bicycle)
Moto (Motorcycle)
Taxi (Taxi)
Helicóptero (Helicopter)
Tren (Train)
Barco (Boat)
Patinete (Scooter)
Camión (Truck)
Autobús (Bus)
Caravana (Caravan)
Furgón/Furgoneta (Van)
Barco velero (Sailboat)

Brócoli (Broccoli)
Mora (Blackberry)
Tortilla (Omelette)
Bellota (Acorn)
Zumo (Juice)
Rábanos (Radish)
Infusión/Té (Tea)
Pera (Pear)
Limón (Lemon)
Piña (Pineapple)
Sandía (Watermelon)
Melón (Melon)
Melocotón (Peach)
Almendras (Almonds)
Zanahoria (Carrot)
Lechuga (Lettuce)
Palomitas (Popcorn)
Aguacate (Avocado)
Coco (Coconut)
Chocolate (Chocolate)
Kiwi (Kiwi)
Sándwich (Sandwich)
Tomate (Tomato)
Cereza (Cherry)
Uvas (Grapes)
Pepinillo (Pickle)
Manzana (Apple)
Cebolla (Onion)
Sopa (Soup)
Berenjena (Eggplant)
Pimiento (Pepper)
Espárrago (Asparagus)
Mandarina (Clementine)
Fresa (Strawberry)
Alcachofa (Artichoke)
Calabacín (Zucchini)
Ciruela (Plum)
Pan (Bread)
Coliflor (Cauliflower)
Café (Coffee)
Agua (Water)
Leche (Milk)
Vino (Wine)
Cerveza (Beer)
Huevo (Egg)
Queso (Cheese)
Arroz (Rice)
Sal (Salt)
Pollo (Chicken)
Ensalada (Salad)
Paella (Paella)
Langostinos (Prawns)
Jamón (Ham)
Helado (Ice cream)
Pescado (Fish)
Chorizo (Chorizo sausage)
Carne (Meat)
Espinacas (Spinach)
Pizza (Pizza)
Bocadillo (Sandwich)
Calamares (Squid)
Chuletas (Chops)
Espagueti (Spaghetti)
Yogur (Yogurt)
Galleta (Cookie)
Alubias (Beans)
Garbanzos (Chickpeas)
Tarta (Cake)
Lentejas (Lentils)

Ojo (Eye)
Nariz (Nose)
Boca/Dientes (Mouth/Teeth)
Oreja (Ear)
Brazo (Arm)
Mano (Hand)
Dedo (Finger)
Codo (Elbow)
Hombros (Shoulders)
Espalda (Back)
Cuello (Neck)
Pierna (Leg)
Rodilla (Knee)
Pies (Feet)
Uña (Nail)
Pelo (Hair)
Ceja (Eyebrow)
Tobillo (Ankle)
Tripa (Belly)
Cadera/Cintura (Hip/Waist)
Cabeza (Head)

Vestido (Dress)
Camiseta (T-shirt)
Camisa (Shirt)
Pantalones (Trousers)
Calcetines (Socks)
Zapatos (Shoes)
Falda (Skirt)
Jersey (Sweater)
Chaqueta (Jacket)
Gorro (Hat)
Abrigo (Coat)
Chaleco (Vest)
Bufanda (Scarf)
Bañador/calzoncillos (Swimsuit/Underwear)
Bañador (Swimsuit)
Bolso (Bag)
Gafas (Glasses)
Sombrero (Hat)
Gorra (Cap)
Collar (Necklace)
Pulsera (Bracelet)
Anillo (Ring)
Pendientes (Earrings)

Libro (Book)
Teléfono (Phone)
Televisión (Television)
Radio (Radio)
Botella (Bottle)
Tenedor (Fork)
Vaso (Glass)
Copa (Wine glass)
Cuchillo (Knife)
Cuchara (Spoon)
Plato (Plate)
Vela (Candle)
Llave (Key)
Balón/Pelota (Ball)
Toalla (Towel)
Jabón (Soap)
Escoba/Cepillo (Broom)
Fregona (Mop)
Peine (Brush)
Bolígrafo (Pen)
Monedas (Coins)
Billetes (Banknotes)
Caja (Box)
Plancha (Iron)
Calculadora (Calculator)
Alicates (Pliers)
Regla (Ruler)
Hacha (Axe)
Enchufe (Plug)
Globo (Balloon)
Cámara (Camera)
Tostadora (Toaster)
Pala (Shovel)
Cacerola/Olla (Pot)
Sartén (Frying pan)
Paraguas (Umbrella)
Silbato/Pito (Whistle)
Sierra/Serrucho (Saw)
Sacapuntas (Sharpener)
Cortauñas (Nail clipper)
Martillo (Hammer)
Tornillo (Screw)
Destornillador (Screwdriver)
Clavo (Nail)
Tijeras (Scissors)
Taladro (Drill)
Grapadora (Stapler)
Linterna (Flashlight)

Sofá (Sofa)
Sillón (Armchair)
Silla (Chair)
Mesa (Table)
Mesilla (Bedside table)
Puerta (Door)
Ventana (Window)
Escaleras (Stairs)
Armario (Wardrobe)
Microondas (Microwave)
Estantería (Shelf)
Lámpara (Lamp)
Nevera/Frigorífico (Fridge)
Cama (Bed)
Cojín (Cushion)
Espejo (Mirror)
Vitrocerámica (Ceramic hob)
Lavabo (Sink)
Horno (Oven)
Ducha (Shower)
Bañera (Bathtub)
Váter/Inodoro (Toilet)

Cine (Cinema)
Cocina (Kitchen)
Baño (Bathroom)
Salón/Comedor (Living/Dining room)
Habitación (Bedroom)
Restaurante/Bar (Restaurant/Bar)
Pescadería (Fish shop)
Carnicería (Butcher’s)
Charcutería (Deli)
Papelería (Stationery shop)
Librería (Bookstore)
Biblioteca (Library)
Peluquería (Hairdresser)
Panadería (Bakery)
Farmacia (Pharmacy)
Teatro (Theatre)
Estanco (Tobacco shop)
Frutería (Greengrocer’s)
Mar (Sea)
Cielo (Sky)
Playa (Beach)
Montaña (Mountain)
Campo (Countryside)
Parque (Park)
Supermercado (Supermarket)

| **Supplementary Table 5**. Summary of Cognitive and Language Assessment Tests in PPA | |
| --- | --- |
| *Cognitive assessment* | |
| Digit Span (Peña – Casanova, 2009) | Forward  Backward |
| Corsi Block-tapping Test (Kaplan et al. 1991) | Forward  Backward |
| Trail Making Test (TMT) (Reitan & Wolfson 1993) | Part A: numbers only  Part B: number and letters |
| Symbol Digit Modalities Test (SDMT) (Smith, 1973) | Number of correct items in 90 seconds |
| Visual Object and Space Perception Battery (VOSP) (Warrington & James, 1991) | Object decision: n of correctly identified objects  Progressive silhouettes: Score based on the stage at which the participant correctly identifies each object as it is progressively revealed from highly degraded to complete form; the final score reflects the last correctly identified item in each series.  Position discrimination: n of items correctly localised.  Number location: Number of items in which the participant correctly matches a spatial position with its corresponding numerical label. |
| Judgement of Line Orientation (JLO) (Benton et al., 1975) | Number of correct items |
| Tower of London Drexel University (ToL) (Culbertson & Zillmer, 2001) | Total correct items, total moves, initiation time (IT), resolution time (RT), execution time = TR - TI |
| Rey Osterrieth Complex Figure Test (Rey, 1941) | Copy accuracy (/36) and time  3 minutes recall (/36)  30 minutes recall (/36)  Recognition (/36) |
| Stroop Test (Golden & Fershwater, 1978) | Word reading: N of words read in 45 seconds.  Colour naming: N of colours named in 45 seconds.  Interference condition: N of colours (incongruent with the written word) named in 45 seconds. |
|  |  |
| *Language assessment* | |
| Reading | Words /24)  Capitalized Words (/24)  Foreign Words (/24)  Non-Words (/24) |
| Initial phoneme omission | Repetition of a word omitting the first phoneme (/12) |
| Word Spelling | Spell 12 words. (/12) |
| Repetition (Test Barcelona, Peña-Casanova et al., 2009) | Words (/10)  Non-words (/8)  Syllables (/8)  Syllable pairs (/8)  Sentences (/60) |
| Semantic Association | Association of pictures with semantic relation (/20). |
| Naming | Objects (/20)  Actions (/20) |
| Matching | Word-Picture: Associate the written word with its picture (/20)  Picture-Action: Associate the spoken verb with its drawn representation (/20) |
| Synonym Judgement | Association of synonyms (/20) |
| Verb tense agreement (/80) | High-frequency 1st conjugation regular verbs (/10)  Low-frequency 1st conjugation regular verbs (/10)  High-frequency 2nd and 3rd conjugation regular verbs (/10)  Low-frequency 2nd and 3rd conjugation regular verbs (/10)  High-frequency 1st conjugation irregular verbs (/10)  Low-frequency 1st conjugation irregular verbs (/10)  High-frequency 2nd and 3rd conjugation irregular verbs (/10)  Low-frequency 2nd and 3rd conjugation irregular verbs (/10)  1st conjugation (/40)  2nd and 3rd conjugation (/40)  Regular verbs (/40)  Irregular verbs (/40)  Frequent verbs (/40)  Infrequent verbs (/40) |
| Complex Sentence comprehension (/20) | Present continuous (/4)  Passive (/4)  Relative (/4)  Object-focused (/4)  Subject-focused (/4)  Non-reversible distractors (number of errors)  Reversible distractors (number of errors) |
| Fluency | Actions: Number of actions named in one minute.  Formal: Number of words starting with P named in one minute.  Semantic: Number of animals named in one minute. |

**Supplementary Table 6.** Brain Metabolism Differences Between Clusters

|  | Cluster 1 | Cluster 2 | U | p |
| --- | --- | --- | --- | --- |
| Left Amygdala | 25.78 | 31.18 | 317.000 | 0.229 |
| Right Amygdala | 28.04 | 29.65 | 369.000 | 0.720 |
| Left Angular Gyrus | 20.87 | 34.50 | 204.000 | **0.002** |
| Right Angular Gyrus | 27.26 | 30.18 | 351.000 | 0.515 |
| Left Calcarine Cortex | 27.57 | 29.97 | 358.000 | 0.591 |
| Right Calcarine Cortex | 29.13 | 28.91 | 388.000 | 0.961 |
| Left Caudate | 24.13 | 32.29 | 279.000 | 0.068 |
| Right Caudate | 25.70 | 31.24 | 315.000 | 0.216 |
| Left Cerebellar Lobule III | 32.48 | 26.65 | 311.000 | 0.193 |
| Right Cerebellar Lobule III | 32.52 | 26.62 | 310.000 | 0.188 |
| Left Cerebellar Lobules IV–V | 33.78 | 25.76 | 281.000 | 0.074 |
| Right Cerebellar Lobules IV–V | 34.43 | 25.32 | 266.000 | 0.042 |
| Left Cerebellar Lobule VI | 34.17 | 25.50 | 272.000 | 0.053 |
| Right Cerebellar Lobule VI | 36.57 | 23.88 | 217.000 | **0.005** |
| Left Cerebellar Lobule VII | 31.74 | 27.15 | 328.000 | 0.305 |
| Right Cerebellar Lobule VII | 34.48 | 25.29 | 265.000 | **0.040** |
| Left Cerebellar Lobule VIII | 36.17 | 24.15 | 226.000 | **0.007** |
| Right Cerebellar Lobule VIII | 34.91 | 25.00 | 255.000 | 0.027 |
| Left Cerebellar Lobule IX | 35.17 | 24.82 | 249.000 | 0.021 |
| Right Cerebellar Lobule IX | 34.09 | 25.56 | 274.000 | 0.057 |
| Left Cerebellar Lobule X | 33.48 | 25.97 | 288.000 | 0.094 |
| Right Cerebellar Lobule X | 32.78 | 26.44 | 304.000 | 0.157 |
| Left Cerebellar Crus I | 32.04 | 26.94 | 321.000 | 0.255 |
| Right Cerebellar Crus I | 35.30 | 24.74 | 246.000 | **0.018** |
| Left Cerebellar Crus II | 32.91 | 26.35 | 301.000 | 0.143 |
| Right Cerebellar Crus II | 34.91 | 25.00 | 255.000 | **0.027** |
| Left Anterior Cingulate | 29.17 | 28.88 | 387.000 | 0.948 |
| Right Anterior Cingulate | 29.09 | 28.94 | 389.000 | 0.974 |
| Left Middle Cingulate | 27.70 | 29.88 | 361.000 | 0.626 |
| Right Middle Cingulate | 29.70 | 28.53 | 375.000 | 0.795 |
| Left Posterior Cingulate | 22.39 | 33.47 | 239.000 | 0.013 |
| Right Posterior Cingulate | 25.57 | 31.32 | 312.000 | 0.199 |
| Left Cuneus | 27.39 | 30.09 | 354.000 | 0.547 |
| Right Cuneus | 28.04 | 29.65 | 369.000 | 0.720 |
| Left Inferior Frontal Gyrus. Opercular Part | 28.35 | 29.44 | 376.000 | 0.807 |
| Right Inferior Frontal Gyrus. Opercular Part | 32.04 | 26.94 | 321.000 | 0.255 |
| Left Inferior Frontal Gyrus. Orbital Part | 29.87 | 28.41 | 371.000 | 0.745 |
| Right Inferior Frontal Gyrus. Orbital Part | 31.39 | 27.38 | 336.000 | 0.371 |
| Left Inferior Frontal Gyrus. Triangular Part | 29.52 | 28.65 | 379.000 | 0.845 |
| Right Inferior Frontal Gyrus. Triangular Part | 32.35 | 26.74 | 314.000 | 0.210 |
| Left Medial Orbital Frontal Cortex | 30.13 | 28.24 | 365.000 | 0.672 |
| Right Medial Orbital Frontal Cortex | 29.43 | 28.71 | 381.000 | 0.871 |
| Left Middle Frontal Gyrus | 29.13 | 28.91 | 388.000 | 0.961 |
| Left Middle Orbital Frontal Cortex | 28.22 | 29.53 | 373.000 | 0.770 |
| Right Middle Orbital Frontal Cortex | 28.70 | 29.21 | 384.000 | 0.909 |
| Right Middle Frontal Gyrus | 30.57 | 27.94 | 355.000 | 0.558 |
| Left Superior Frontal Gyrus | 29.43 | 28.71 | 381.000 | 0.871 |
| Left Superior Medial Frontal Gyrus | 32.13 | 26.88 | 319.000 | 0.242 |
| Right Superior Medial Frontal Gyrus | 32.22 | 26.82 | 317.000 | 0.229 |
| Left Superior Orbital Frontal Gyrus | 28.91 | 29.06 | 389.000 | 0.974 |
| Right Superior Orbital Frontal Gyrus | 31.13 | 27.56 | 342.000 | 0.425 |
| Right Superior Frontal Gyrus | 31.57 | 27.26 | 332.000 | 0.337 |
| Left Fusiform Gyrus | 25.13 | 31.62 | 302.000 | 0.148 |
| Right Fusiform Gyrus | 30.35 | 28.09 | 360.000 | 0.614 |
| Left Heschl’s Gyrus | 20.39 | 34.82 | 193.000 | **0.001** |
| Right Heschl’s Gyrus | 23.04 | 33.03 | 254.000 | **0.026** |
| Left Hippocampus | 22.70 | 33.26 | 246.000 | 0.018 |
| Right Hippocampus | 27.43 | 30.06 | 355.000 | 0.558 |
| Left Insula | 26.22 | 30.88 | 327.000 | 0.298 |
| Right Insula | 28.30 | 29.47 | 375.000 | 0.795 |
| Left Lingual Gyrus | 28.26 | 29.50 | 374.000 | 0.782 |
| Right Lingual Gyrus | 31.09 | 27.59 | 343.000 | 0.435 |
| Left Inferior Occipital Gyrus | 26.57 | 30.65 | 335.000 | 0.362 |
| Right Inferior Occipital Gyrus | 29.65 | 28.56 | 376.000 | 0.807 |
| Left Middle Occipital Gyrus | 26.78 | 30.50 | 340.000 | 0.407 |
| Right Middle Occipital Gyrus | 29.13 | 28.91 | 388.000 | 0.961 |
| Left Superior Occipital Gyrus | 27.87 | 29.76 | 365.000 | 0.672 |
| Right Superior Occipital Gyrus | 30.00 | 28.32 | 368.000 | 0.708 |
| Left Olfactory Cortex | 28.35 | 29.44 | 376.000 | 0.807 |
| Right Olfactory Cortex | 28.65 | 29.24 | 383.000 | 0.896 |
| Left Pallidum | 29.83 | 28.44 | 372.000 | 0.757 |
| Right Pallidum | 32.57 | 26.59 | 309.000 | 0.182 |
| Left Paracentral Lobule | 27.13 | 30.26 | 348.000 | 0.484 |
| Right Paracentral Lobule | 30.22 | 28.18 | 363.000 | 0.649 |
| Left Parahippocampal Gyrus | 32.13 | 26.88 | 319.000 | 0.242 |
| Right Parahippocampal Gyrus | 34.39 | 25.35 | 267.000 | **0.044** |
| Left Inferior Parietal Lobule | 23.61 | 32.65 | 267.000 | **0.044** |
| Right Inferior Parietal Lobule | 29.70 | 28.53 | 375.000 | 0.795 |
| Left Superior Parietal Lobule | 26.91 | 30.41 | 343.000 | 0.435 |
| Right Superior Parietal Lobule | 29.00 | 29.00 | 391.000 | 1.000 |
| Left Postcentral Gyrus | 28.09 | 29.62 | 370.000 | 0.733 |
| Right Postcentral Gyrus | 31.26 | 27.47 | 339.000 | 0.398 |
| Left Precentral Gyrus | 28.57 | 29.29 | 381.000 | 0.871 |
| Right Precentral Gyrus | 33.43 | 26.00 | 289.000 | 0.097 |
| Left Precuneus | 25.91 | 31.09 | 320.000 | 0.248 |
| Right Precuneus | 28.74 | 29.18 | 385.000 | 0.922 |
| Left Putamen | 28.30 | 29.47 | 375.000 | 0.795 |
| Right Putamen | 29.30 | 28.79 | 384.000 | 0.909 |
| Left Gyrus Rectus | 30.61 | 27.91 | 354.000 | 0.547 |
| Right Gyrus Rectus | 30.09 | 28.26 | 366.000 | 0.684 |
| Left Rolandic Operculum | 24.70 | 31.91 | 292.000 | 0.107 |
| Right Rolandic Operculum | 29.26 | 28.82 | 385.000 | 0.922 |
| Left Supplementary Motor Area | 32.74 | 26.47 | 305.000 | 0.162 |
| Right Supplementary Motor Area | 34.30 | 25.41 | 269.000 | **0.047** |
| Left Supramarginal Gyrus | 22.43 | 33.44 | 240.000 | **0.014** |
| Right Supramarginal Gyrus | 28.74 | 29.18 | 385.000 | 0.922 |
| Left Inferior Temporal Gyrus | 22.35 | 33.50 | 238.000 | **0.013** |
| Right Inferior Temporal Gyrus | 27.87 | 29.76 | 365.000 | 0.672 |
| Left Middle Temporal Gyrus | 22.43 | 33.44 | 240.000 | **0.014** |
| Right Middle Temporal Gyrus | 26.30 | 30.82 | 329.000 | 0.313 |
| Left Middle Temporal Pole | 28.87 | 29.09 | 388.000 | 0.961 |
| Right Middle Temporal Pole | 29.83 | 28.44 | 372.000 | 0.757 |
| Left Superior Temporal Pole | 26.48 | 30.71 | 333.000 | 0.345 |
| Right Superior Temporal Pole | 29.17 | 28.88 | 387.000 | 0.948 |
| Left Superior Temporal Gyrus | 23.87 | 32.47 | 273.000 | 0.055 |
| Right Superior Temporal Gyrus | 29.48 | 28.68 | 380.000 | 0.858 |
| Left Thalamus | 25.26 | 31.53 | 305.000 | 0.162 |
| Right Thalamus | 25.91 | 31.09 | 320.000 | 0.248 |
| Vermis Lobules I–II | 30.13 | 28.24 | 365.000 | 0.672 |
| Vermis Lobule III | 31.78 | 27.12 | 327.000 | 0.298 |
| Vermis Lobules IV–V | 35.52 | 24.59 | 241.000 | **0.015** |
| Vermis Lobule VI | 37.78 | 23.06 | 189.000 | **0.001** |
| Vermis Lobule VII | 34.43 | 25.32 | 266.000 | **0.042** |
| Vermis Lobule VIII | 31.78 | 27.12 | 327.000 | 0.298 |
| Vermis Lobule IX | 34.04 | 25.59 | 275.000 | 0.059 |
| Vermis Lobule X | 33.43 | 26.00 | 289.000 | 0.097 |
| Vermis | 35.43 | 24.65 | 243.000 | **0.016** |

Note: Bold values indicate statistically significant differences between clusters (p < 0.05). *U= Mann–Whitney U test

**Supplementary Table 7.** Coefficients of LASSO and Elastic Net analysis for MLSE

|  | LASSO | Elastic Net |
| --- | --- | --- |
| *Total* R^2^(RMSE) | 0.52 (4.94) | 0.57 (5.05) |
| Treatment | **3.73** | **3.49** |
| MLSE Total | -0.042 | -0.0206 |
| Digit Span Backward | **0.53** | **0.454** |
| Stroop Word | 0.012 | 0.058 |
| Stroop Colour | **0.22** | **0.197** |
| VOSP Progressive silhouettes | **0.13** | **0.134** |
| VOSP Position discrimination | -2.306e^-4^ | 0.0258 |
| Reading of Foreign Words | 4.009e^-4^ | - |
| CSC (Relative) | 0.0017 | 0.058 |
| Repetition of Syllables | **0.39** | **0.376** |
| Repetition of Sentences | 0.09 | 0.077 |
| Right Cerebellum | **0.15** | **0.121** |
| Right Posterior Cingulate | **0.103** | **0.107** |
| Left Hippocampus | **0.115** | 0.092 |
| Right Insula | 0.034 | 0.042 |
| Left Superior Temporal Pole | 9.189e^-4^ | 0.004 |
| Right Hippocampus | - | 0.007 |
| Naming Words | - | 5.412e^-4^ |
| Left Cerebellum | - | 0.008 |
| Right Cerebellum | - | -0.019 |
| *Active group* R^2^(RMSE) | 0.218 (4.28) | 0.137 (4.38) |
| Stroop Colour Baseline | **0.235** | **0.238** |
| CSC (Reversible distractors) | 0.016 | 0.051 |
| Left Inferior Occipital Gyrus | 0.019 | 0.027 |
| Digit Span Backward | - | 0.043 |
| Left Superior Medial Frontal Gyrus | - | -7.74e^-4^ |
| Left Fusiform Gyrus | - | 0.002 |
| Right Fusiform Gyrus | - | 0.016 |
| *Sham group* R^2^(RMSE) | 0.685 (5.61) | 0.137 (4.38) |
| Digit Span Forward Baseline | **1.327** | **0.986** |
| CSC (Object focused) | **0.136** | **0.283** |
| Repetition of Words | **3.282** | **2.25** |
| Repetition of Sentences | 0.033 | 0.045 |
| Right Heschl's Gyrus | 0.049 | 0.039 |
| Digit Span Backward | - | 0.035 |
| Repetition of minimal pairs | - | 0.179 |
| Right Superior Temporal Pole | - | 0.017 |

Note: Variables in bold are those with absolute coefficients greater than 0.1. Abbreviations: MLSE= Mini Lingüistic State Examination; VOSP= Visual Object and Space Perception Battery; CSC= Complex Sentence Comprehension.

**Supplementary Table 8.** Coefficients of LASSO and Elastic Net analysis for Naming

|  | LASSO | Elastic Net |
| --- | --- | --- |
| *Total* R^2^(RMSE) | 0.422 (15.0) | 0.41 (15.17) |
| Treatment | **13.351** | **11.444** |
| ROCF (Time) | 0.046 | **0.123** |
| VOSP (Number location) | **-0.158** | **-0.198** |
| Left Cerebellum | -0.072 | **-0.151** |
| Right Cerebellum | **-0.272** | **-0.190** |
| Left Posterior Cingulate, | **0.180** | **0.208** |
| Right Superior Orbital Frontal Gyrus | **0.352** | **-0.279** |
| Left Hippocampus | **0.445** | **0.387** |
| Left Inferior Occipital Gyrus | 0.098 | 0.088 |
| Vermis | -0.008 | -0.052 |
| Reading of Foreign Words | - | -6.054e^-4^ |
| Right Inferior Frontal Gyrus (Triangular Part) | - | -0.0489 |
| *Active group* R^2^(RMSE) |  |  |
| Left Cerebellum | -9.885e^-16^ |  |

Note: Variables in bold are those with absolute coefficients greater than 0.1. Abbreviations: ROCF= Rey–Osterrieth Complex Figure; VOSP= Visual Object and Space Perception Battery.

**Supplementary Table 9.** Coefficients of LASSO and Elastic Net analysis for IDDD

|  | LASSO | Elastic Net |
| --- | --- | --- |
| *Total* R^2^(RMSE) | 0.462 (6.12) | 0.58 (5.35) |
| Treatment | **-2.779** | **-2.727** |
| IDDD Baseline | **-0.221** | **-0.233** |
| Corsi Span Forward | -0.014 | **-0.101** |
| Corsi Span Backward | -0.006 | **-0.138** |
| ROCF (3min recall) | **-0.185** | **-0.148** |
| ROCF (30min recall) | **-0.104** | **-0.191** |
| ROCF (Recognition) | -0.096 | **-0.147** |
| VOSP (Object decision) | **-0.219** | **-0.272** |
| VTA (High-frequency 1st conjugation regular verbs) | **0.102** | -0.081 |
| Left Rolandic Operculum | 0.0261 | -0.039 |
| NPI Total | - | -0.019 |
| Education (years) | - | 0.066 |
| ROCF (Recognition) | - | -0.014 |
| ToL (Total correct items) | - | 0.086 |
| CSC (non-reversible distractors) | - | **-0.135** |
| VTA (Low-frequency 1st conjugation regular verbs) | - | -0.005 |
| Repetition of Non-Words | - | **0.214** |
| Repetition of minimal pairs | - | 0.098 |
| Left Heschl's Gyrus | - | -0.0302 |
| Right Heschl's Gyrus | - | -0.0065 |
| *Active group* R^2^(RMSE) | *0.156 (7.20)* | *0.16 (7.3)* |
| IDDD Baseline | -0.068 | **-0.102** |
| ROCF (3min recall) | **-0.204** | - |
| Repetition of Non-Words | 0.0746 | 0.0461 |
| *Sham group* R^2^(RMSE) | 0 (6.62) | 0 |
| Repetition of Syllables | 3.396e^-16^ |  |

Note: Variables in bold are those with absolute coefficients greater than 0.1. Abbreviations: IDDD= Interview for Deterioration in Daily Living Activities in Dementia; NPI= Neuropsychiatric Inventory; ROCF= Rey–Osterrieth Complex Figure; VOSP= Visual Object and Space Perception Battery; VTA: Verb Tense Agreement; ToL= Tower of London; CSC= Complex Sentence Comprehension.

**Supplementary Table 10.** Coefficients of LASSO and Elastic Net analysis for NPI

|  | LASSO | Elastic Net |
| --- | --- | --- |
| *Total* R^2^(RMSE) | 0.807 (3.47) | 0.769 (3.79) |
| Treatment | **-3.851** | **-3.318** |
| PPA Variant | **-0.652** | **-0.513** |
| NPI Total | **-0.352** | **-0.311** |
| Stroop Word | 0.019 | 0.015 |
| Stroop Interference | 0.067 | 0.042 |
| VOSP (Object decision) | -0.021 | -0.005 |
| VOSP Position discrimination | **0.409** | **0.321** |
| CSC (Subject focused) | **-1.479** | **-1.229** |
| CSC (Relative) | -0.006 | -0.095 |
| CSC (Non-reversible distractors) | **0.168** | **0.151** |
| Repetition of pairs of syllables | **0.341** | **0.344** |
| Repetition of Words | **0.429** | **0.564** |
| Age | -0.025 | -0.022 |
| Right Caudate | **-0.171** | **-0.142** |
| Right Cerebellum | 0.052 | 0.048 |
| Left Paracentral Lobule | 0.057 | 0.041 |
| Right Superior Parietal Lobule | -0.014 | -0.034 |
| Right Supramarginal Gyrus | 0.062 | -0.075 |
| Right Inferior Temporal Gyrus | -0.168 | -0.076 |
| Left Middle Temporal Gyrus | 0.078 | 0.067 |
| Left Superior Temporal Gyrus | **0.162** | **0.151** |
| Vermis Lobules I–II | 0.038 | 0.037 |
| Vermis Lobules VIII | -5.362e^-4^ | - |
| ROCF (Time) | - | 6.376e^-4^ |
| Right Middle Temporal Pole | - | -0.022 |
| *Active group* R^2^(RMSE) | 0.865 (3.09) | 0.96 (1.46) |
| NPI Total | **-0.417** | **-0.488** |
| IDDD Baseline | 0.005 | 0.035 |
| ACE-III Language | 0.037 | 0.086 |
| ROCF (Recognition) | -0.009 | **-0.240** |
| Stroop Word | 0.022 | 0.035 |
| Stroop Interference | 0.041 | **0.232** |
| Word-Picture Matching | **-0.215** | **-0.185** |
| CSC (Subject focused) | **-1.298** | **-1.092** |
| CSC (Non-reversible distractors) | **0.068** | **0.251** |
| Repetition of pairs of syllables | **0.255** | **0.549** |
| Repetition of Words | 0.013 | 0.051 |
| Repetition of Sentences | 0.051 | 0.039 |
| Age | -0.084 | **-0.161** |
| Right Angular Gyrus | -0.057 | - |
| Right Caudate | **-0.145** | **-0.160** |
| Right Inferior Frontal Gyrus (Triangular Part) | -0.012 | - |
| Right Fusiform Gyrus | **-0.176** | - |
| Left Heschl's Gyrus | 0.084 | **0.109** |
| Left Superior Temporal Gyrus | **0.232** | **0.181** |
| Vermis Lobules I–II | 0.084 | 0.004 |
| Synonym Judgement | - | **0.178** |
| Initial phoneme omission | - | 0.029 |
| Right Cuneus | - | -0.0905 |
| Left Amygdala | - | 0.0779 |
| Left Calcarine Cortex | - | 0.023 |
| Left Cerebellar Lobules IV–V | - | ,-0.004 |
| Right Cerebellar Lobule IX | - | 0.039 |
| Right Fusiform Gyrus | - | **-0.198** |
| Right Paracentral Lobule | - | -0.005 |
| Right Parahippocampal Gyrus | - | 0.027 |
| Right Inferior Temporal Gyrus | - | -0.002 |
| Vermis Lobules VIII | - | -0.085 |
| Digit Span Backward | - | **-0.247** |
| Corsi Span Forward | - | -0.08 |
| TMT (B) | - | 0.002 |
| ROCF (Copy) | - | 0.082 |
| *Sham group* R^2^(RMSE) | 0.068 (5.04) | 0.94 (1.174) |
| ROCF (Time) | 0.001 | 0.007 |
| NPI Total | - | **-0.104** |
| ROCF (Recognition) | - | **0.452** |
| VOSP Number location | - | **-0.174** |
| CSC (Present Continuous) | - | **-0.493** |
| Repetition of Words | - | **-0.149** |
| Right Cerebellar Crus I | - | 0.067 |
| Right Cerebellar Crus II | - | 0.081 |
| Left Posterior Cingulate | - | 0.015 |
| Right Gyrus Rectus | - | **-0.137** |
| Right Middle Temporal Pole,- | - | -0.037 |
| Left Thalamus | - | **0.232** |

Note: Variables in bold are those with absolute coefficients greater than 0.1. Abbreviations: PPA= Primary Progressive Aphasia; IDDD= Interview for Deterioration in Daily Living Activities in Dementia; NPI= Neuropsychiatric Inventory; ROCF= Rey–Osterrieth Complex Figure; VOSP= Visual Object and Space Perception Battery; VTA: Verb Tense Agreement; ToL= Tower of London; CSC= Complex Sentence Comprehension; ACE-III= Addenbrooke's Cognitive Examination – Third Edition; TMT= Trail Making Test.

**Supplementary Table 11.** Coefficients of LASSO and Elastic Net analysis for WPM

|  | LASSO | Elastic Net |
| --- | --- | --- |
| *Total* R^2^(RMSE) | 0.19 (16.7) | 0.19 (16.65) |
| CSC (Passive) | **-1.880** | **-1.638** |
| Right Amygdala | 0.004 | **0.118** |
| Left Superior Occipital Gyrus | **-0.285** | **-0.241** |
| Stroop Word | - | -0.004 |
| Left Superior Parietal Gyrus | - | -0.036 |
| *Active group* R^2^(RMSE) | 0 | 0 |
| Left Superior Occipital Gyrus | -1.67e^-16^ | - |
| *Sham group* R^2^(RMSE) | 0 | 0 |
| WPM | -2.594e^-4^ | - |
| Right Anterior Cingulate Gyrus | **0.462** | - |
| Left Middle Temporal Gyrus | **-0.714** | - |

Note: Variables in bold are those with absolute coefficients greater than 0.1. Abbreviations: CSC= Complex Sentence Comprehension; WPM= Word per Minute.

**Supplementary Table 12.** Correlations between clinical variables

|  | MLSE | Naming | WPM | IDDD | NPI |
| --- | --- | --- | --- | --- | --- |
| MLSE | - | 0.379* | -0.011 | -0.359* | -0.248 |
| Naming | 0.379* | - | -0.003 | -0.440* | -0.065 |
| WPM | -0.011 | -0.003 | - | -0.108 | -0.148 |
| IDDD | -0.359* | -0.440* | -0.108 | - | 0.212 |
| NPI | -0.248 | -0.065 | -0.148 | 0.212 | - |

*Correlation is significant *(p* < 0.05)

**Supplementary Table 13.** Clinical and Demographic Characteristics of the Active TMS Group Across Clusters at Baseline.

|  | Cluster 1 | Cluster 2 | U | p |
| --- | --- | --- | --- | --- |
| Sex (Female) | 6 (66.7%) | 30 (75%) | 0.430 | 0.512 |
| Age | 75.77 | 70.29 | 85.000 | 0.077 |
| IDDD Total | 47.33 (10.82) | 47.32 (14.32) | 139.500 | 1.000 |
| MLSE Total | 77.22 (10.75) | 79.68 (12.42) | 115.500 | 0.437 |
| SUVR Baseline | 0.79 (0.08) | 0.84 (0.08) | 88.000 | 0.095 |
| Naming | 123.56 (71.96) | 160.70 (72.00) | 91.500 | 0.120 |
| WPM | 71.23 (39.14) | 70.14 (27.71) | 116.000 | 0.970 |
| NPI Total | 13.33 (15.04) | 10.57 (10.23) | 122.500 | 0.580 |
| ACE-III Total | 47.67 (16.37) | 19.52 (20.53) | 93.000 | 0.132 |
| ACE-III Attention | 11.44 (3.807) | 13.45 (4.06) | 84.500 | 0.073 |
| ACE-III Memory | 8.67 (6.24) | 13.26 (7.271) | 77.000 | **0.043** |
| ACE-III Fluency | 4.56 (2.08) | 4.77 (3.16) | 135.000 | 0.883 |
| ACE-III Language | 13.22 (5.88) | 14.84 (6.15) | 116.500 | 0.455 |
| ACE-III Visuospatial | 9.78 (3.25) | 12.06 (3.731) | 74.500 | **0.034** |
| Digit Span (Forward) | 4.33 (1.51) | 4.52 (1.351) | 120.500 | 0.524 |
| Digit Span (Backward) | 2.33 (1.204) | 2.81 (1.35) | 106.500 | 0.264 |
| Corsi Blocks (Forward) | 4.11 (1.21) | 4.42 (1.63) | 108.500 | 0.302 |
| Corsi Blocks (Backward) | 2.44 (1.701) | 3.35 (1.624) | 87.000 | 0.080 |
| TMT-A | 142.78 (176.34) | 114.00 (148.82) | 108.500 | 0.315 |
| TMT-B | 702.56 (336.31) | 504.06 (349.1) | 99.000 | 0.165 |
| SDMT | 11.67 (12.37) | 19.74 (12.34) | 86.500 | 0.086 |
| ROCF Copy | 20.17 (11.42) | 25.00 (10.47) | 102.500 | 0.230 |
| ROCF 3-min Recall | 5.556 (5.23) | 11.177 (6.91) | 76.500 | **0.041** |
| ROCF 30-min Recall | 5.278 (5.78) | 9.806 (7.08) | 85.500 | 0.080 |
| ROCF Recognition | 16.67 (5.7) | 17.65 (5.3) | 91.000 | 0.110 |
| Stroop Word | 40.22 (20.91) | 47.71 (20.76) | 117.000 | 0.466 |
| Stroop Colour | 21.33 (15.34) | 27.29 (14.64) | 100.500 | 0.206 |
| Stroop Interference | 8.67 (11.94) | 13.10 (11.61) | 104.500 | 0.253 |
| ToL Total Correct | 1.22 (1.79) | 1.71 (2.13) | 117.500 | 0.440 |
| ToL Total Moves | 4.67 (76.23) | 5.97 (82.17) | 111.000 | 0.324 |
| VOSP Object Decision | 14.33 (3.8) | 15.55 (4.31) | 90.500 | 0.110 |
| VOSP Progressive Silhouettes | 14.44 (5.08) | 12.74 (4.68) | 104.500 | 0.255 |
| VOSP Position Discrimination | 18.00 (1.95) | 18.71 (3.68) | 106.000 | 0.252 |
| VOSP Number Location | 7.56 (3.37) | 7.29 (3.4) | 126.000 | 0.656 |
| Semantic Fluency (Animals) | 5.33 (2.67) | 7.03 (5.01) | 115.000 | 0.425 |
| Formal Fluency (P) | 7.67 (3.61) | 6.45 (3.79) | 104.500 | 0.254 |
| JLO | 13.11 (11.27) | 16.68 (9.54) | 107.500 | 0.298 |
| Reading Words | 21.56 (3.76) | 21.45 (6.09) | 139.000 | 0.986 |
| Reading Capital Letters | 20.56 (4.47) | 18.58 (6.11) | 110.000 | 0.335 |
| Reading Foreign Words | 10.22 (7.91) | 13.68 (8.27) | 105.000 | 0.262 |
| Reading Non-Words | 18.78 (6.96) | 18.35 (6.88) | 130.000 | 0.756 |
| Initial Phoneme Omission | 7.00 (4.17) | 6.52 (4.54) | 131.000 | 0.781 |
| Word Spelling | 3.00 (3.61) | 4.00 (3.87) | 123.000 | 0.587 |
| Repetition of Non-Words | 6.78 (3.81) | 5.61 (3.5) | 116.000 | 0.444 |
| Semantic Association | 19.11 (1.14) | 18.65 (2.09) | 129.000 | 0.711 |
| Object Naming | 7.11 (6.06) | 11.03 (6.31) | 89.000 | 0.101 |
| Word–Picture Matching | 19.22 (0.86) | 19.55 (1.21) | 105.500 | 0.147 |
| Synonym Judgement | 18.44 (4.98) | 14.90 (6.15) | 88.000 | 0.092 |
| Action Naming | 13.11 (5.26) | 15.87 (4.34) | 83.500 | 0.065 |
| Picture–Action Matching | 18.22 (2.46) | 18.68 (2.46) | 130.500 | 0.746 |
| Complex Sentence Comprehension (Total) | 11.33 (5.44) | 13.13 (5.76) | 112.000 | 0.372 |
| Verb Tense Agreement Total | 37.22 (34.02) | 49.39 (31.99) | 121.500 | 0.555 |
| Action Fluency | 7.67 (5.07) | 7.71 (4.5) | 134.500 | 0.871 |
| Repetition of Syllable | 7.33 (1.93) | 6.90 (1.32) | 126.000 | 0.640 |
| Repetition of Syllable Pair | 6.78 (2.58) | 6.23 (2.31) | 119.500 | 0.494 |
| Repetition of Non-Word | 5.11 (2.82) | 5.16 (2.93) | 124.500 | 0.620 |
| Repetition of Minimal Pairs | 5.67 (2.39) | 5.19 (2.68) | 132.500 | 0.818 |
| Repetition of Word | 8.89 (1.27) | 9.23 (1.42) | 120.500 | 0.469 |
| Repetition of Sentence | 45.56 (18.64) | 46.13 (15.78) | 125.500 | 0.649 |

Note: Values are presented as mean (SD), unless otherwise indicated. Bold values indicate statistically significant differences between clusters (p < 0.05). Abbreviations: IDDD= Interview for Deterioration in Daily Living Activities in Dementia; MLSE= Mini Lingüistic State Examination; SDMT= Symbol Digit Modalities Test; NPI= Neuropsychiatric Inventory; ROCF= Rey–Osterrieth Complex Figure; VOSP= Visual Object and Space Perception Battery; ToL= Tower of London; ACE-III= Addenbrooke's Cognitive Examination – Third Edition; TMT= Trail Making Test; WPM= Word per Minute. JLO: Benton Judgment of Line Orientation test; U= Mann–Whitney U test.

**Supplementary Figure 1.** Distribution of baseline outcome measures

**Supplementary Figure 1.** Distribution of baseline outcome measures**.** Histograms illustrating the distribution of baseline values for the primary and secondary outcome measures included in the study: SUVR, Naming, MLSE, WPM, IDDD, and NPI. The number of participants was 57 for all measures except WPM (N=50). The x-axis represents the value of each outcome measure, and the y-axis represents the frequency of participants. Abbreviations: SUVR= Standardized Uptake Value Ratio; IDDD= Interview for Deterioration in Daily Living Activities in Dementia; MLSE= Mini Linguistic State Examination; NPI= Neuropsychiatric Inventory; WPM= Words per Minute.

**Supplementary Material Reference list:**

-Benton AL, Hannay HJ, Varney N. Visual perception of line direction in patients with unilateral brain disease. Neurology 1974: 25, 907–910.

-Culbertson WC, Zillmer EA. Tower of London. Drexel University. TOLDX. North Tonawanda, NY: Multi-Health Systems, 2001.

-Golden CJ. Stroop Color and Word Test. Chicago, IL: Stoeling, 1978.

-Gorno-Tempini, ML, Hillis AE, Weintraub S, Kertesz A, Mendez M, Cappa SF, ... & Grossman, M. Classification of primary progressive aphasia and its variants. Neurology 2011;76: 1006-1014.

-Kaplan, E., Fein, D., Morris, R., & Delis, D. WAIS-R as a neuropsychological instrument. San Antonio, TX: The Psychological Corporation, 1991.

-Peña-Casanova, J. Programa Integrado de Exploración Neuropsicologica. Test Barcelona-Revisado [Integrated program of neuropsychological assessment—Revised Barcelona Test]. Barcelona: Masson, 2005.

-Reitan, R. M., & Wolfson, D. The Halstead-Reitan neuropsychological test battery. Theory and clinical interpretation (2nd. ed.). Tucson, AZ: Neuropsychology Press, 1993.

-Rey A. L’examen psychologique dans les cas d’encéphalopathie traumatique. Archives de Psychologie 1941, 28 : 286–340.

- Smith A. Symbol Digit Modalities Test Manual. Los Angeles: Western Psychological Services, 1973.

-Warrington, E. K., & James, M. The visual object and space perception battery. Bury St. Edmunds, Suffolk, England: Thames Valley Test Company, 1991.
